# Endothelin signalling mediates experience-dependent myelination in the CNS

**DOI:** 10.7554/eLife.49493

**Published:** 2019-10-28

**Authors:** Matthew Swire, Yuri Kotelevtsev, David J Webb, David A Lyons, Charles ffrench-Constant

**Affiliations:** 1 MRC Centre for Regenerative Medicine, MS Society Edinburgh CentreUniversity of EdinburghEdinburghUnited Kingdom; 2 Centre for Discovery Brain SciencesUniversity of EdinburghEdinburghUnited Kingdom; 3 Centre for Neurobiology and Brain RestorationSkoltech Institute for Science and TechnologyMoscowRussian Federation; 4 British Heart Foundation Centre of Research Excellence, Centre of Cardiovascular Science, Queen's Medical Research InstituteUniversity of EdinburghEdinburghUnited Kingdom; Max Planck Institute of Experimental MedicineGermany; Texas Children's HospitalUnited States

**Keywords:** oligodendrocytes, myelination, endothelin, Mouse, Rat, Zebrafish

## Abstract

Experience and changes in neuronal activity can alter CNS myelination, but the signalling pathways responsible remain poorly understood. Here we define a pathway in which endothelin, signalling through the G protein-coupled receptor endothelin receptor B and PKC epsilon, regulates the number of myelin sheaths formed by individual oligodendrocytes in mouse and zebrafish. We show that this phenotype is also observed in the prefrontal cortex of mice following social isolation, and is associated with reduced expression of vascular endothelin. Additionally, we show that increasing endothelin signalling rescues this myelination defect caused by social isolation. Together, these results indicate that the vasculature responds to changes in neuronal activity associated with experience by regulating endothelin levels, which in turn affect the myelinating capacity of oligodendrocytes. This pathway may be employed to couple the metabolic support function of myelin to activity-dependent demand and also represents a novel mechanism for adaptive myelination.

## Introduction

There is increasing evidence that experience regulates CNS myelination. For example, social interactions, sensory stimulation and several forms of learning have been shown to alter white matter and myelin structure in both humans and animal models (Scholz et al., 2009; Makinodan et al., 2012; Liu et al., 2012; Sampaio-Baptista et al., 2013; McKenzie et al., 2014; Etxeberria et al., 2016; Xiao et al., 2016; Hughes et al., 2018). At the level of individual neurons and axons, increasing the level of activity by using optogenetics or chemogenetics enhances the generation of myelin-forming oligodendrocytes and increases the amount of myelin they form (Gibson et al., 2014; Mitew et al., 2018), whilst preventing synaptic vesicular release from axons reduces myelin formation (Hines et al., 2015; Mensch et al., 2015; Koudelka et al., 2016). Together, these findings have led to a new concept of CNS plasticity - adaptive myelination. This concept posits that changes in neuronal activity in response to experience of the extrinsic environment lead to local changes in myelination. Such changes could in turn contribute to the alterations in conduction that underpin CNS neural circuit plasticity (Sampaio-Baptista and Johansen-Berg, 2017; Foster et al., 2019; Suminaite et al., 2019).

Exploring this important concept requires that we understand the mechanisms that can link changes in neuronal activity to the regulation of myelination. In addition to the communication between axons of active neurons and the oligodendrocytes that myelinate them, neuronal activity might also affect myelination indirectly in the local area through signals from other glial cells or from vascular cells that can respond to dynamic changes in neuronal activity. Such indirect signalling has been implied by in vitro studies, wherein increased levels of LIF secreted by cultured astrocytes in the presence of neuronal activity enhances myelination (Ishibashi et al., 2006). However, whether the vasculature might relay information about neuronal activity and in turn influence myelination by oligodendrocytes in vivo is not known.

Here we set out to test the hypothesis that the vasoactive peptide endothelin (EDN) enables blood vessels to play such a role in indirectly linking activity and myelination. This hypothesis is based on two sets of prior data. First, EDN expression by endothelial cells increases with enhanced blood flow (Yanagisawa et al., 1988; Dancu et al., 2004; Walshe et al., 2005; Pandit et al., 2015), which occurs in response to increased CNS activity. Second, the EDN G-protein coupled receptor (GPCR) endothelin receptor B (EDNRB) enhances myelination in slice cultures (Yuen et al., 2013). Here we show that EDN is expressed by CNS vascular cells, and that this expression lessens in the medial prefrontal cortex following social isolation, which we confirm also leads to impaired cortical myelination. Correspondingly, we show, by manipulating EDNRB signalling in rodent and zebrafish models, that reduced EDN signalling decreases the number of myelin sheaths formed by individual oligodendrocytes in vivo. Finally, we rescue the reduction in myelination associated with environmental social deprivation by intranasal administration of an EDNRB agonist to activate EDN signalling within the CNS. Together our data indicate that the vasculature responds to environmental signals associated with changes in neuronal activity and can, in turn, affect the myelinating capacity of oligodendrocytes in vivo. In this way, we propose a novel mode by which active neurons may regulate oligodendrocyte behaviour and provide a mechanism for adaptive myelination.

## Results

### The levels of EDN expression in blood vessels are reduced following social isolation

If EDN signalling from CNS blood vessels provides a link between circuit function and myelination, then an initial prediction would be that EDN expression by these vessels will be responsive to extrinsic environmental changes that alter neuronal activity and myelination. To test this prediction, we used a model in which the extrinsic environment has been shown to regulate CNS myelination: social isolation (Liu et al., 2012; Makinodan et al., 2012).

**Figure 1.**
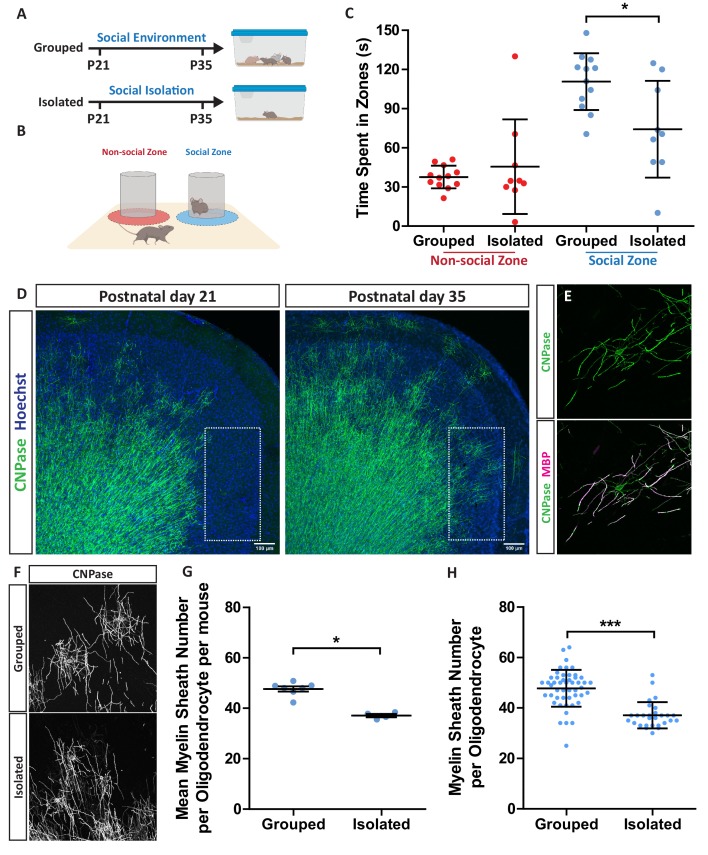
Social isolation in mice reduces layer II/III medial prefrontal cortex oligodendrocyte myelin sheath number. (**A**) Timeline for social isolation experiment. At postnatal day 21 male mice were housed in a social environment containing 3–5 mice or on their own in isolation. Mice were analysed at P35. (**B**) Schematic of social interaction assay. Mice were recorded for 5 min exploring an arena containing two identical wire mesh containers: one container housed an unrelated male wild type mouse (social zone), while the other remained empty (non-social zone). (**C**) Time spent within 2.5 cm of non-social container: Grouped 37.58 s ± 8.683 n = 12, Isolated 45.59 ± 36.28 n = 9 and social container: Grouped 110.7 s ± 21.71 n = 12, Isolated 74.24 s ± 37.07 n = 9 (mean ± standard deviation). Unpaired T-test p=0.0107. (**D**) Coronal section of mouse prefrontal cortex stained for CNPase and nuclei. Layers II/III of the medial prefrontal cortex outlined by dashed box. Scale bars = 100 μm. (**E**) Layer II/III oligodendrocyte stained for CNPase and MBP. (**F**) Representative images of medial prefrontal cortex oligodendrocytes stained for CNPase. (**G**) Mean number of myelin sheaths formed by oligodendrocytes per mouse. Grouped 47.66 ± 1.015 n = 7 mice, Isolated 37.11 ± 0.6425 n = 4 mice (mean ± standard error). Mann-Whitney test, p=0.0106. (**H**) Pooled data for number of myelin sheaths formed by layer II/III medial prefrontal cortex oligodendrocytes. Grouped 47.80 ± 7.289 n = 49 cells from seven mice, Isolated 37.11 ± 5.202 n = 28 cells from four mice (mean ± standard deviation). Mann-Whitney test, p=<0.001.

Prior work has established that social isolation in mice during a critical period comprising 2 weeks after weaning reduces both the excitability of specific subtypes of pyramidal neurons of the medial prefrontal cortex (mPFC) and oligodendrocyte formation and myelination in the same area (Liu et al., 2012; Makinodan et al., 2012; Yamamuro et al., 2018). We repeated this protocol, confirming the previously-described effect on circuit function by showing that isolated mice spent significantly less time than socially-experienced controls interacting with a novel mouse (Figure 1A–C). To quantify the myelination defects in these mice immediately following the isolation period we used a labelling strategy in which oligodendrocyte cell bodies, processes and myelin sheaths are revealed by CNPase immunostaining. The sparsely-myelinated layers II/III of the mPFC, a region myelinated during this isolation period (Figure 1D), enabled individual oligodendrocyte morphologies to be analysed for myelin sheath number and length (Figure 1D–E, Figure 1—figure supplement 1). Using this approach we showed that individual oligodendrocytes made fewer myelin sheaths (Figure 1F–H), although the length of the myelin sheaths formed by oligodendrocytes in these mice was unaffected (Figure 1—figure supplement 2E–F). Further, we found that the isolated mice also generated fewer oligodendrocytes in the mPFC (Figure 1—figure supplement 2C–D). In contrast, social isolation had no effect on myelin sheath number in the visual cortex (Figure 1—figure supplement 3A–B), demonstrating the region-dependent effects of this protocol on myelination.

**Figure 1—figure supplement 1.**
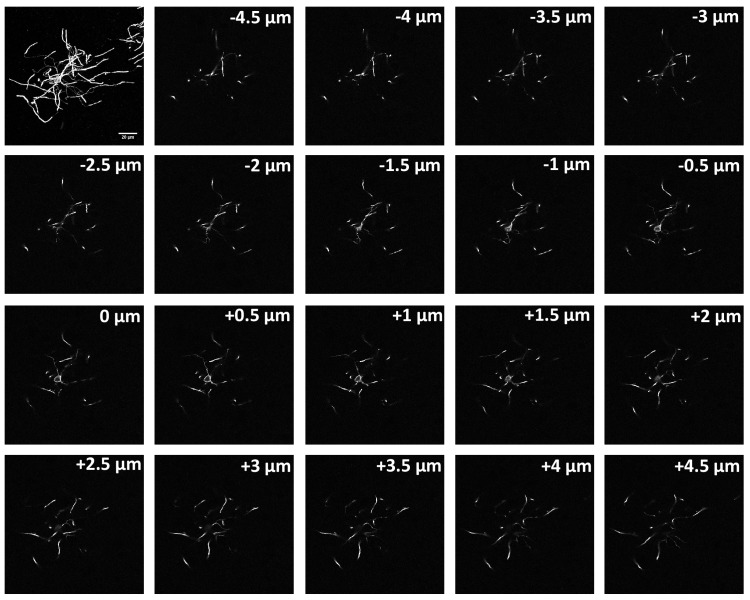
Z-stack through a CNPase positive oligodendrocyte. A full maximum intensity projection of a complete a layer II/III oligodendrocytes in mouse cortex stained for CNPase followed by 0.5 µm slices. Tracing the fine processes through each slice enables assessment of the myelin sheaths formed by individual oligodendrocytes.

**Figure 1—figure supplement 2.**
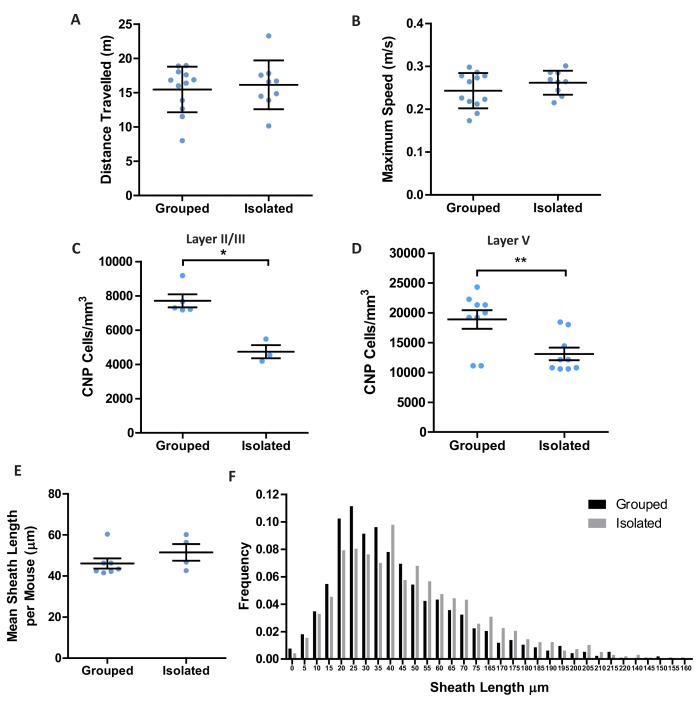
Social isolation reduces oligodendrocyte generation in the mPFC. (**A**) Distance travelled by mice during 5 min of exploration of the social interaction assay. Grouped 15.48 meters ± 3.326 n = 12 mice, Isolated 16.15 meters ± 3.57 n = 9 mice (mean ± standard deviation). (**B**) Maximum speed travelled by mice during 5 min of exploration of the social interaction assay. Grouped 0.2433 ± 0.04117 n = 12 mice, Isolated 0.2619 meters ± 0.02792 n = 9 mice (mean ± standard deviation). (**C**) Quantification of CNP positive cells in medial prefrontal cortex layers II/III: Grouped 7709 ± 378.7 n = 5 mice, Isolated 4738 ± 385.7 n = 4 mice (mean ± standard error). (**D**) Quantification of CNP positive cells in medial prefrontal cortex layer V: Grouped 18879 ± 1559 n = 9 mice, Isolated 13099 ± 1052 n = 9 mice (mean ± standard error). (**E**) Mean myelin sheath length formed by oligodendrocytes per mouse. Grouped 46.10 µm ± 2.485 n=seven mice, Isolated 51.48 µm ± 4.08 n=four mice (mean ± standard error). (**F**) Frequency distribution of myelin sheath lengths.

**Figure 1—figure supplement 3.**
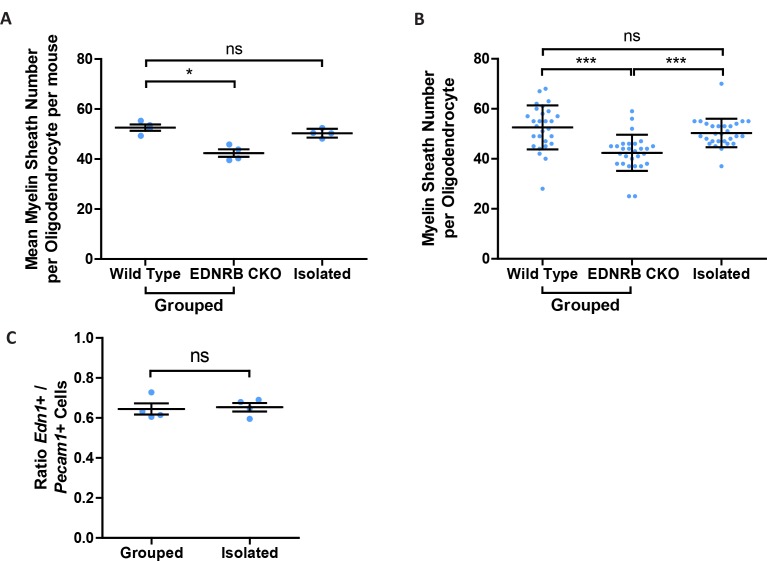
Loss of oligodendroglial EDNRB reduces myelin sheath number in the visual cortex where social isolation does not affect myelination or *Edn1* expression. (**A**) Mean number of myelin sheath formed by oligodendrocytes per mouse. Wild type 52.57 ± 1.270 n = 4 mice, EDNRB CKO 42.39 ± 1.487 n = 4 mice, Isolated 50.32 ± 0.8755 n = 4 mice (mean ± standard error). Mann-Whitney test, p=0.0106. Kruskal-Wallis test, with Dunns post hoc. (**B**) Pooled data for number of myelin sheaths formed by layer II/III visual cortex oligodendrocytes. Grouped 52.57 ± 8.779 n = 28 cells from four mice, EDNRB CKO 42.39 ± 7.213 n = 28 cells from four mice, Isolated 50.32 ± 5.716 n = 28 cells from four mice (mean ± standard deviation). Kruskal-Wallis test, with Dunns post hoc. (**C**) Quantification of the number of *Edn1* expressing *Pecam1* positive cells. Grouped 0.6449 ± 0.02814 n = 4 mice, Isolated 0.63535 ± 0.02120 n = 4 mice (mean ± standard error). Mann-Whitney test.

**Figure 2.**
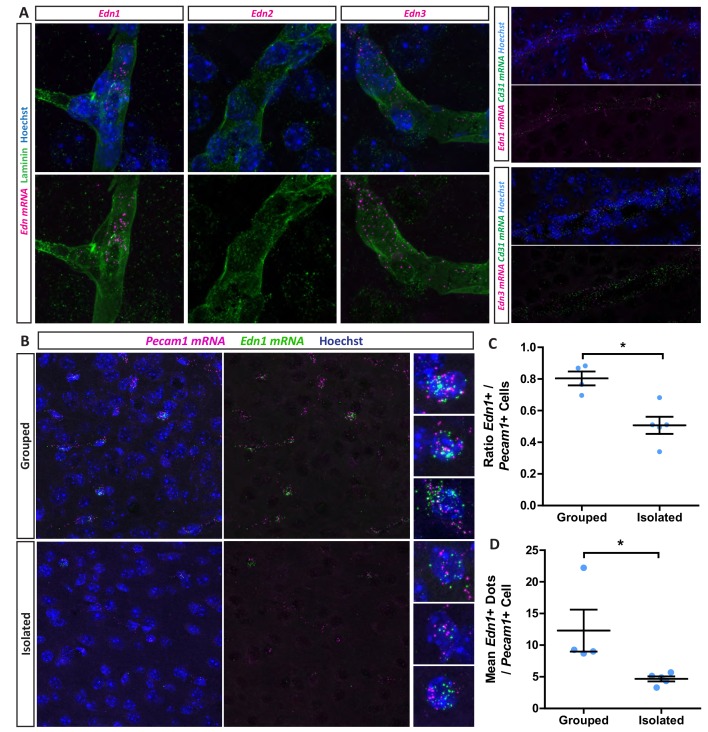
Social isolation reduces vascular endothelin expression. (**A**) *Edn1* and *Edn3* mRNA expression in laminin positive and *CD31* positive blood vessels as revealed by RNAScope in situ hybridisation. (**B**) Representative images of *Edn1* and *Pecam1* mRNA expression in the mPFC. (**C**) Quantification of the number of *Edn1* expressing *Pecam1* positive endothelial cells. Grouped 0.8033 ± 0.04411 n = 4 mice, Isolated 0.5074 ± 0.05412 n = 5 mice (mean ± standard error). Mann-Whitney test, p=0.0159. (**D**) Quantification of the mean *Edn1* mRNA molecules expressed by *Pecam1* positive cells per mouse. Grouped 12.29 ± 3.312 n = 4 mice, Isolated 4.673 ± 0.4059 n = 5 mice (mean ± standard error). Mann-Whitney test, p=0.0159.

Having validated our social isolation protocol by demonstrating experience-dependent changes in myelination, we next analysed EDN expression in the mPFC. We performed in situ hybridization studies examining the expression of all three EDN ligands (*Edn1-3*) in controls and following isolation. We confirmed robust *Edn1* and *Edn3* mRNA expression in controls, localised to laminin-positive blood vessels, which co-expressed the endothelial/pericyte marker *Pecam1* mRNA (Figure 2A) (Yanagisawa et al., 1988; Hammond et al., 2014). While previous studies have observed *Edn1* expression also localised to astrocytes following demyelination (Hammond et al., 2014) and microglia (Zhang et al., 2014), here we observed no *Edn1*, *Edn2* or *Edn3* mRNA in S100β positive astrocytes or IBA1 positive microglia (Figure 2—figure supplement 1A–B), indicating that endothelial cells and/or pericytes are the main source of *Edn* in the healthy mouse brain. Following social isolation, both the number of endothelial cells expressing *Edn1* and *Edn3* mRNA and the expression level of *Edn1* mRNA within each cell was significantly reduced in the mPFC (Figure 2B–D, Figure 2—figure supplement 2F–G). However, the vascular area and the number of cells expressing *Pecam1* mRNA were unaffected (Figure 2—figure supplement 2A–E). In contrast to the mPFC, there was no effect of social isolation on *Edn1* expression by *Pecam1* cells in the visual cortex (Figure 1—figure supplement 3C). We therefore conclude that the environmental deprivation associated with social isolation reduces vascular *Edn* production in the mouse mPFC.

**Figure 2—figure supplement 1.**
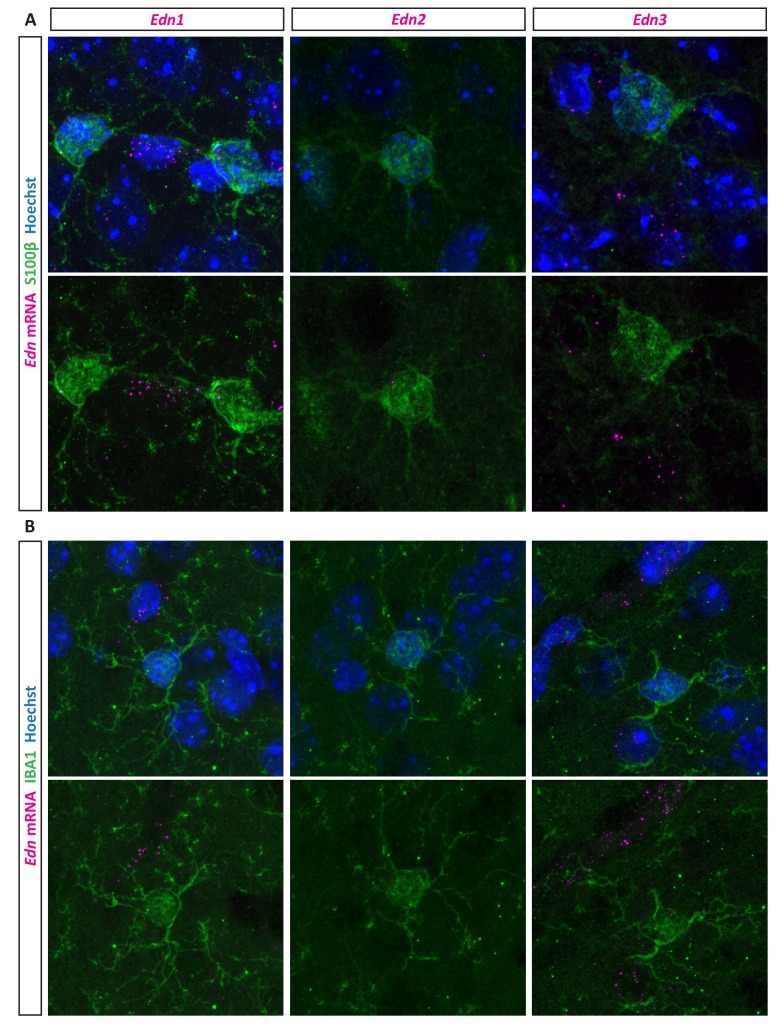
EDN mRNA is not expressed in astrocytes and microglia. Expression of *Edn1*, *Edn2* and *Edn3* in S100β positive astrocytes and IBA1 positive microglia. (**A**) RNAScope in situ hybridisation for *Edn1*, *Edn2* and *Edn3* mRNA in the mouse medial prefrontal cortex stained for S100β positive astrocytes. Note that S100β positive cells are negative for *Edn1* and *Edn3* mRNA while positive *Edn* signal can be seen in S100β negative cells. (**B**) RNAScope in situ hybridisation for *Edn1*, *Edn2* and *Edn3* mRNA in the mouse medial prefrontal cortex stained for Iba1 positive microglia. Note that Iba1 positive cells are negative for *Edn1 *and *Edn3 *mRNA while positive *Edn* signal can be seen in Iba1 negative cells.

**Figure 2—figure supplement 2.**
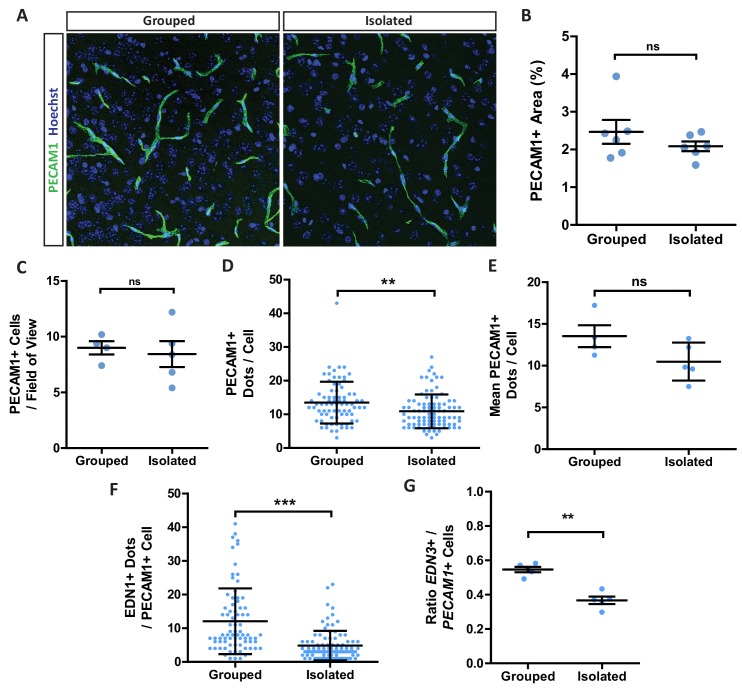
Social isolation does not affect medial prefrontal cortex vasculature. (**A**) Representative images of medial prefrontal cortex vasculature staining for PECAM1. (**B**) Quantification of PECAM1 area in medial prefrontal cortex layer II/III: Grouped 2.468% ± 0.3156 n = 6 mice, Isolated 2.086 ± 0.13 n = 6 mice (mean ± standard error). Mann-Whitney test. (**C**) Quantification of number of *Pecam1* mRNA expressing cells per field of view. Grouped 9 ± 0.5888 n = 4 mice, Isolated 8.44 ± 1.162 n = 4 mice (mean ± standard error). Mann-Whitney test. (**D**) Quantification of the number of *Pecam1* mRNA molecules. Grouped 13.47 ± 6.204 n = 77 cells from four mice, Isolated 10.89 ± 5.02 n = 92 cells from five mice (mean ± standard deviation). Mann-Whitney test, p=0.002. (**E**) Quantification of the number of *Pecam1* mRNA molecules per mouse. Grouped 13.53 ± 1.305 n = 4 mice, Isolated 10.48 ± 1.018 n = 5 mice (mean ± standard error). Mann-Whitney test. (**F**) Quantification of the number of *Edn1* mRNA molecules in *Pecam1 *positive endothelial cells. Grouped 12.08 ± 9.759 n = 77 cells from four mice, Isolated 4.837 ± 4.406 n = 92 from five mice (mean ± standard deviation). Mann-Whitney test, p<0.0001. (**G**) Quantification of the number of *Edn3* expressing *Pecam1* positive endothelial cells. Grouped 0.5463 ± 0.01586 n = 5 mice, Isolated 0.3674 ± 0.02163 n = 5 mice (mean ± standard error). Mann-Whitney test, p=0.0079.

### EDNRB loss reduces the number of myelin sheaths formed by individual oligodendrocytes in vivo

**Figure 3.**
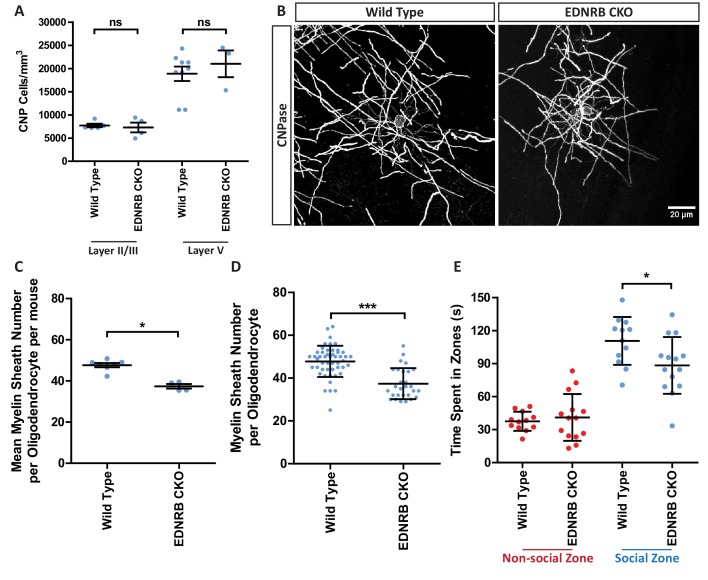
Loss of oligodendroglial EDNRB reduces myelin sheath number and reduces sociability. (**A**) Quantification of CNP positive cells in medial prefrontal cortex layers II/III: Wild type 7709 ± 378.7 n = 5 mice, EDNRB CKO 7288 ± 1054 n = 4 mice (mean ± standard error) and layer V: Wild type 18879 ± 1559 n = 9 mice, EDNRB CKO 21016 ± 2878 n = 3 mice (mean ± standard error). Mann Whitney test, layer II/III p=0.9048, layer V p=0.3527. (**B**) Representative images of medial prefrontal cortex oligodendrocytes stained for CNPase. Scale bar = 20 μm. (**C**) Mean number of myelin sheath formed by oligodendrocytes per mouse. Wild type 47.66 ± 1.015 n = 7 mice, EDNRB CKO 37.39 ± 1.099 n = 4 mice (mean ± standard error). Mann-Whitney test, p=0.0106. (**D**) Pooled data for number of myelin sheaths formed by layer II/III medial prefrontal cortex oligodendrocytes. Wild type 47.80 ± 7.289 n = 49 cells from seven mice, EDNRB CKO 37.39 ± 7.208 n = 28 cells from four mice (mean ± standard deviation). Mann-Whitney test, p=<0.001. (**E**) Time spent within 2.5 cm of non-social container: Wild type 37.58 s ± 8.683 n = 12, EDNRB CKO 41.06 ± 21.29 n = 14 and social container: Wild type 110.7 s ± 21.71 n = 12, EDNRB CKO 88.39 s ± 25.79 n = 14 (mean ± standard deviation). Unpaired T-test p=0.0267.

Next, we asked whether a reduction in EDN signalling could potentially contribute to the reduced levels of oligodendrocyte generation and myelination in the isolated mice. To do this, we defined the role of EDN signalling in myelination by performing genetic loss of function experiments to remove the relevant receptor in conditional knockout mice. We targeted the EDN receptor EDNRB due to the high levels of expression in forebrain oligodendrocytes and our own previous work identifying EDNRB as a regulator of myelination in vitro (Yuen et al., 2013; Horiuchi et al., 2017; Marques et al., 2018). We generated a conditional knock out (cKO) using a floxed *Ednrb* mouse line crossed with a line expressing cre recombinase driven by the *Pdgfra* promoter to delete *Ednrb* from oligodendrocytes and their precursor cells through development (Figure 3—figure supplement 1A). Comparison of the number of oligodendrocyte lineage cells in the mPFC in the control (*Pdgfra*-cre;*Ednrb*^wt/wt^) mice shown in Figure 1 with EDNRB cKO (*Pdgfra*-cre;*Ednrb*^flox/flox^) mice revealed no significant difference in the oligodendrocyte progenitor cell (OPC) or oligodendrocyte generation (Figure 3A, Figure 3—figure supplement 1B–D). However, loss of EDNRB in the oligodendroglial lineage significantly reduced the number of myelin sheaths generated by individual oligodendrocytes (Figure 3B–D). EDNRB cKO oligodendrocytes demonstrated a 22% reduction in myelin sheath number compared to wild type littermates, but the lengths of the remaining sheaths were unchanged (Figure 3—figure supplement 1E–F). A similar effect of EDNRB loss on myelin sheath number was also seen in the visual cortex (Figure 1—figure supplement 3A–B). These experiments show that oligodendroglial EDNRB regulates the number of myelin sheaths formed by individual oligodendrocytes and, strikingly, that this effect of deleting EDNRB was identical to the myelination deficits of individual oligodendrocytes within the mPFC following social isolation in wild-type animals.

**Figure 3—figure supplement 1.**
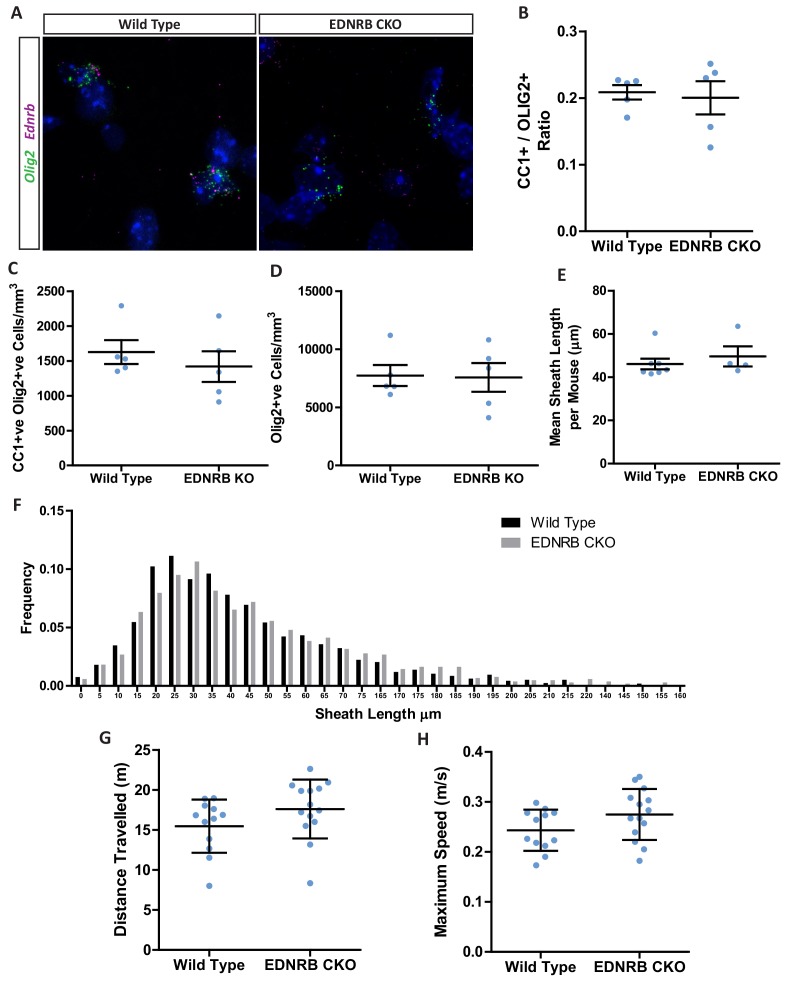
Conditional EDNRB knock out does not affect oligodendrocyte generation or myelin sheath length. (**A**) Representative Images of *Ednrb* mRNA expressing *Olig2* mRNA positive oligodendroglia. (**B**) Ratio of CC1 positive oligodendrocytes over total population of Olig2 positive oligodendroglia. Wild type 0.2086 ± 0.01087 n = 5 mice, EDNRB CKO 0.2003 ± 0.0248 n = 5 mice (mean ± standard error). (**C**) Number of CC1 and Olig2 positive oligodendrocytes. Wild type 1626 ± 170.6 n = 5 mice, EDNRB CKO 1419 ± 220.5 n = 5 mice (mean ± standard error). (**D**) Number Olig2 positive oligodendroglia. Wild type 7737 ± 903.8 n = 5 mice, EDNRB CKO 7565 ± 1239 n = 5 mice (mean ± standard error). (**E**) Mean myelin sheath length formed by oligodendrocytes per mouse. Wild type 46.10 µm ± 2.485 n=seven mice, EDNRB CKO 49.64 µm ± 4.688 n=four mice (mean ± standard error). (**F**) Frequency distribution of myelin sheath lengths. (**G**) Distance travelled by mice during 5 min of exploration of the social interaction assay. Wild type 15.48 meters ± 3.326 n = 12 mice, EDNRB CKO 17.62 meters ± 3.677 n = 9 mice (mean ± standard deviation). (**H**) Maximum speed travelled by mice during 5 min of exploration of the social interaction assay. Wild type 0.2433 ± 0.04117 n = 12 mice, EDNRB CKO 0.2748 meters ± 0.05095 n = 14 mice (mean ± standard deviation).

By contrast, the numbers of oligodendrocytes within the mPFC differs between the two experimental manipulations – reduced by social isolation (Figure 1—figure supplement 2C–D) but not affected by deleting oligodendroglial EDNRB. Given these similarities and difference in the cellular phenotypes of social isolation and EDNRB deletion, we therefore next asked to what extent the changes in behaviour following isolation were phenocopied in the EDNRB cKO mice. Surprisingly, despite the additional effects of social isolation on oligodendrocyte number, the cell type specific loss of EDNRB from the oligodendrocyte lineage was sufficient to cause a significant reduction in the amount of time that cKO mice spent with the novel mouse in the social interaction task, although, as might have been expected, the effect size was smaller than in socially isolated mice, with a 33% reduction following social isolation and 20% following EDNRB cKO as compared to wild type mice (Figure 3E, Figure 3—figure supplement 1G–H). We conclude that loss of EDNRB signalling in oligodendrocyte lineage cells phenocopies a specific component of the response of oligodendrocytes to social isolation and that this, in turn, contributes to the behavioural deficits that result from social isolation.

### EDNRB signalling enhances myelin sheath number in 3D microfiber cultures

**Figure 4.**
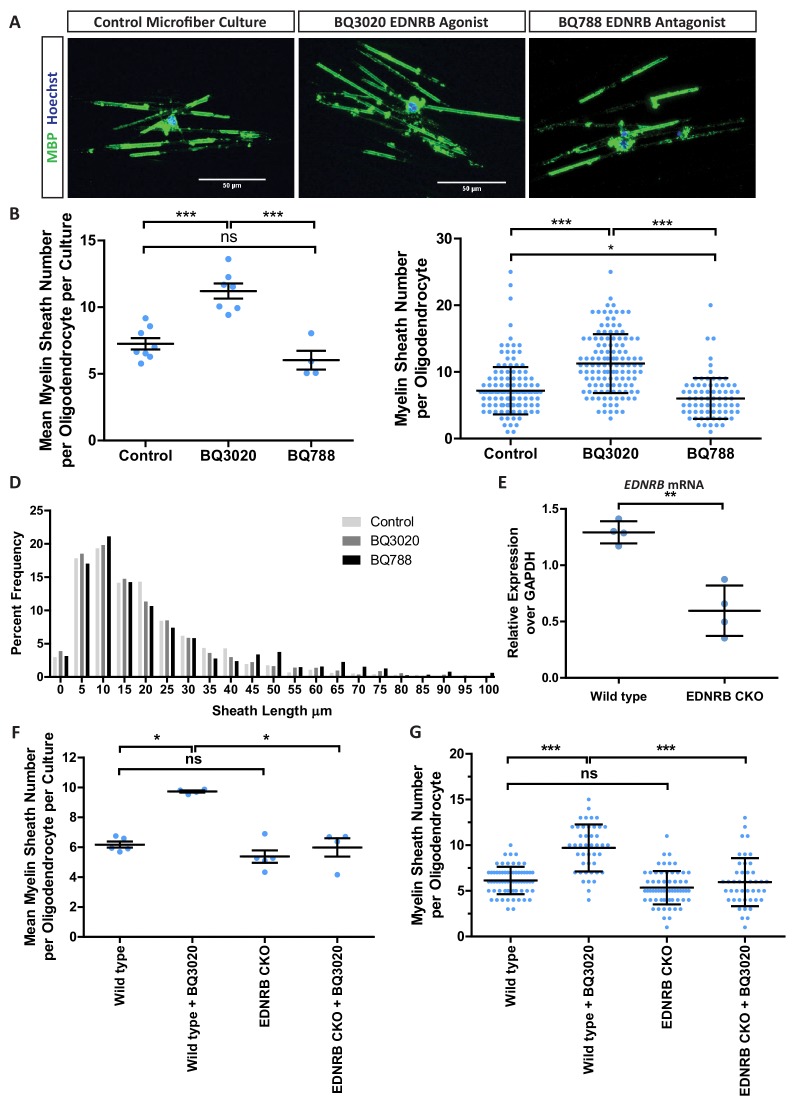
EDNRB enhances myelin sheath number in vitro. (**A**) Representative images of MBP positive oligodendrocytes in microfiber culture. Scale bar = 50 μm. (**B**) Mean number of myelin sheaths formed by rat oligodendrocytes on microfibers per independent culture preparation. Control 7.253 ± 0.4258 n = 8 independent cultures, BQ3020 11.21 ± 0.58635 n = 7 independent cultures, BQ788 6.026 ± 0.7046 (mean ± standard error) n = 4 independent cultures. 1-way ANOVA with Tukey’s post hoc test. (**C**) Pooled data for number of myelin sheaths formed by rat oligodendrocytes on microfibers. Control 7.194 ± 3.544 n = 160 cells from eight independent cultures, BQ3020 11.25 ± 4.420 n = 127 from seven independent cultures, BQ788 6.024 ± 3.059 n = 85 cells from four independent cultures (mean ± standard deviation). Kruskal-Wallis test, with Dunns post hoc. (**D**) Frequency distribution of myelin sheath lengths formed on microfibers. (**E**) qPCR for EDNRB from mouse oligodendrocyte cultures. Wild type 1.292 ± 0.04933 n = 4 independent cultures, EDNRB CKO 0.5958 ± 0.1117 n = 4 independent cultures, BQ788 6.026 ± 0.7046 (mean ± standard error). Unpaired T-test p=0.0013. (**F**) Mean number of myelin sheaths formed by mouse oligodendrocytes on microfibers per independent culture preparation. Wild type 6.18 ± 0.2082 n = 5 independent cultures, Wild type + BQ3020 9.732 ± 0.07548 n = 4 independent cultures, EDNRB CKO 5.380 ± 0.4181 n = 5 independent cultures, EDNRB CKO + BQ3020 5.989 ± 0.6125 n = 4 independent cultures (mean ± standard error). 1-way ANOVA. (**G**) Pooled data for number of myelin sheaths formed by mouse oligodendrocytes on microfibers. Wild type 6.138 ± 1.499 n = 65 cells from five independent cultures, Wild type + BQ3020 9.705 ± 2.575 n = 44 from 4independent cultures, EDNRB CKO 5.345 ± 1.824 n = 65 cells from 5independent cultures, EDNRB CKO + BQ3020 5.955 ± 2.632 n = 44 cells from four independent cultures (mean ± standard deviation). Kruskal-Wallis test, with Dunns post hoc.

Given that cell-type specific loss of EDNRB results in oligodendrocytes making fewer myelin sheaths per cell, we wanted to ask whether activation of EDNRB signalling in oligodendrocytes might have the complementary effect and promote myelin sheath generation. To do this, we turned to a reductionist in vitro system in which myelination takes place in the absence of confounding influences from other cell types - a 3-dimensional microfiber culture system in which oligodendrocytes form myelin sheaths (Lee et al., 2012a; Bechler et al., 2015). In these cultures we tested both a peptide agonist (BQ3020) and a small molecule antagonist (BQ788) of the receptor. Rat oligodendrocyte precursors were seeded onto poly-l-lactic acid-coated microfibers and allowed to differentiate for 3 days before being treated with BQ3020 or BQ788. We saw a significant increase in the number of myelin sheaths produced by individual oligodendrocytes 11 days later in response to the EDNRB agonist (Figure 4A–C). Similar results were obtained with wild type mouse oligodendrocytes (*Pdgfra*-cre;*Ednrb*^wt/wt^) while BQ3020 had no effect on EDNRB cKO (*Pdgfra*-cre;*Ednrb*^flox/flox^) oligodendrocytes (Figure 4F–G), confirming the specificity of the agonist for EDNRB. In contrast to the agonist, treatment with the EDNRB antagonist had no effect on myelin sheath number in these cultures (Figure 4A–C), as might be expected given the lack of EDN-expressing blood vessel cells, and neither agonist nor antagonist altered sheath length (Figure 4D). We conclude from these experiments that the BQ3020 agonist selectively activates EDNRB-mediated signalling pathways in oligodendrocytes that increase myelin sheath number in vitro.

### EDNRB promotes myelin sheath formation through Protein Kinase C ε

**Figure 5.**
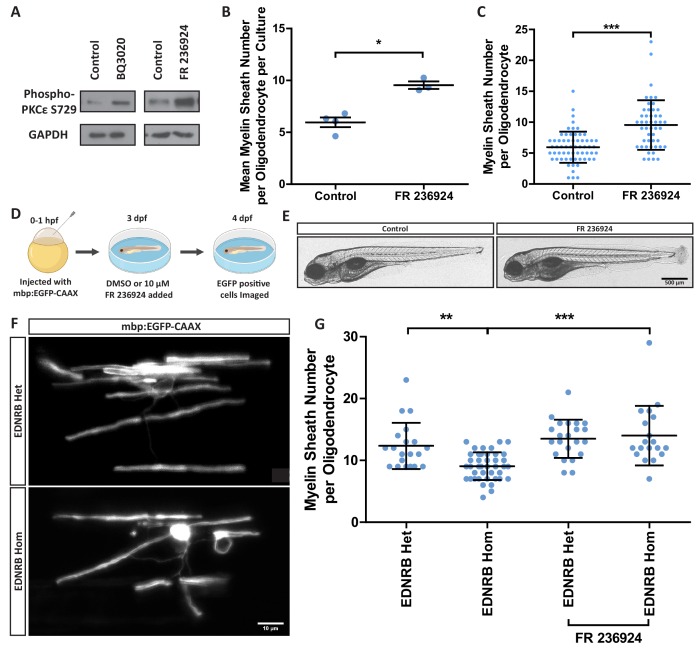
Protein kinase C epsilon is downstream of EDNRB to regulate myelin sheath number. (**A**) Western blot images of rat oligodendrocytes treated with EDNRB agonist BQ3020 and PKCε agonist FR 236924 for 15 min. Antibodies used: Phosphorylated PKCε S729 and loading control GAPDH. (**B**) Mean number of myelin sheaths formed by rat oligodendrocytes on microfibers per experiment. Control 5.959 ± 0.4708 n = 4, FR 236924 9.542 ± 0.3614 n = 3 (mean ± standard error). Unpaired T-test p=0.0024. (**C**) Pooled data for number of myelin sheaths formed by rat oligodendrocytes on microfibers. Control 5.952 ± 2.525 n = 62 cells from four experiments, FR 236924 9.542 ± 4.016 n = 48 from three experiments (mean ± standard deviation). Mann-Whitney test, p=<0.001. (**D**) Schematic for zebrafish larvae treatment with FR 236924. (**E**) Representative images of 4 dpf zebrafish larvae treated with DMSO control or FR 236924. Scale bar = 500 μm. (**F**) Representative images of mbp:EGFP-CAAX oligodendrocytes in four dpf zebrafish larvae. Scale bar = 10 μm. (**G**) Pooled data for number of myelin sheaths formed by zebrafish oligodendrocytes. EDNRB Het 12.35 ± 3.746 n = 20 cells, EDNRB Hom (*rse*) 9.073 ± 2.229 n = 41 from four experiments, EDNRB Het + FR 236924 13.5 ± 3.098 n = 22 cells from five experiments, EDNRB Hom + FR 236924 14 ± 4.807 n = 19 cells (mean ± standard deviation). 1-way ANOVA.

The observation that we can stimulate myelination by oligodendrocytes in an EDNRB-dependent manner in vitro enabled a biochemical approach to identify the downstream signalling mechanisms by which this was mediated. We therefore next used a forward-phase phosphorylated-protein antibody array of mouse oligodendroglial cultures treated with the EDNRB agonist as an initial screen to identify these downstream pathways. As shown in supplementary file 1, one of the largest changes in protein phosphorylation following EDNRB activation was an increase in the phosphorylation of serine-729 of PKC epsilon, an isozyme of the Protein Kinase C (PKC) family. We confirmed an instructive role of PKCε in two ways. First, we examined myelination in oligodendrocyte microfiber cultures treated with FR 236924, a specific activator of PKCε, which promoted phosphorylation of PKCε at serine-729 (Figure 5A) and significantly increased myelin sheath formation (Figure 5B–C), similarly to BQ3020. Second, we used zebrafish, due to their amenability for rapid pharmacological treatment and assessment, to test the prediction that the effects on myelination caused by the loss of EDNRB signalling would be rescued by activating the downstream target PKCε in vivo. We obtained a zebrafish line containing a mutation termed rose (*rse*) in one of the orthologues of *Ednrb (ednrba)*. Analysis of individual oligodendrocytes enabled by sparse labelling of these cells in the fish larvae revealed the same phenotype as in the EDNRB cKO mice; the average number of myelin sheaths formed per oligodendrocyte was reduced by 31% in *rse* homozygous fish (EDNRB Hom) compared to wild type controls (Figure 5F–G, Figure 5—figure supplement 2A), while sheath length was unaffected (Figure 5—figure supplement 2B–C). As predicted, activation of PKCε by FR 236924 treatment from 3 to 4 days post fertilisation, rescued myelin sheath number in *rse* homozygous fish (Figure 5D–G). This result confirms a role for PKCε downstream of EDNRB in the regulation of myelin sheath number in vivo.

**Figure 5—figure supplement 1.**
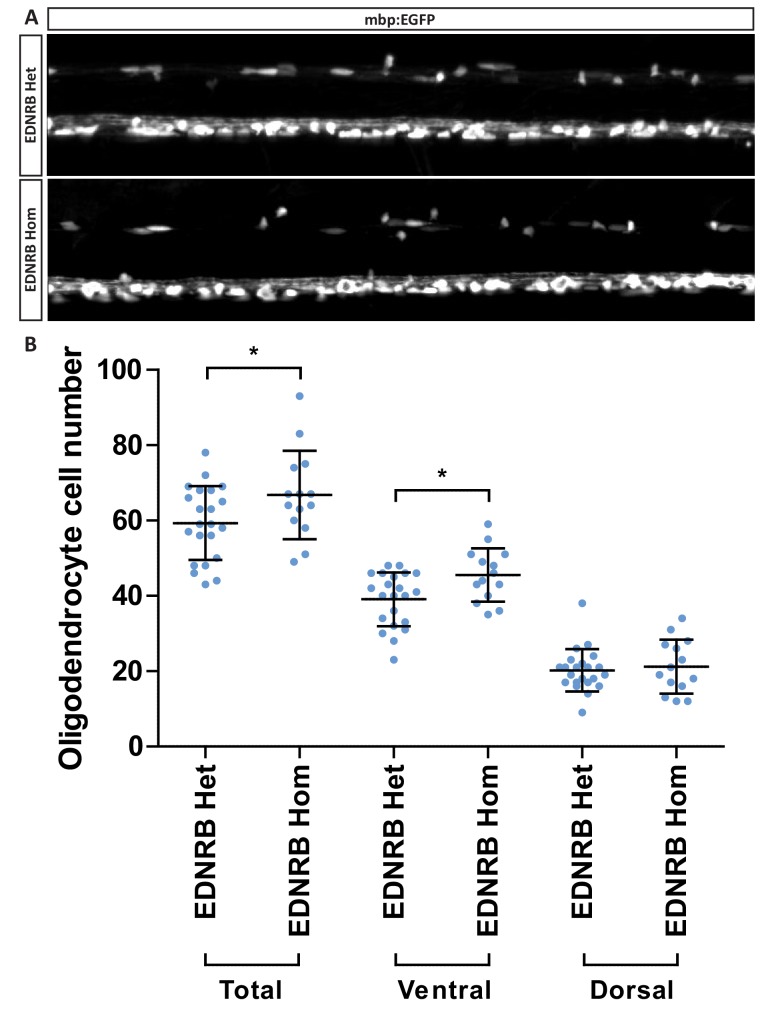
Global loss of EDNRB increases the number of oligodendrocytes in the zebrafish ventral spinal cord. (**A**) Representative images of MBP positive oligodendrocytes in the zebrafish spinal cord. (**B**) Oligodendrocyte cell numbers in the dorsal tract: EDNRB Het 20.23 ± 5.639, EDNRB Hom 21.21 ± 7.138, ventral tract: EDNRB Het 39.09 ± 7.157, EDNRB Hom 45.47 ± 7.09 and total: EDNRB Het 59.32 ± 9.766, EDNRB Hom 66.79 ± 11.73. EDNRB Het n = 22, EDNRB Hom n = 14. T-test, Ventral p=0.0119, Total p=0.0463.

**Figure 5—figure supplement 2.**
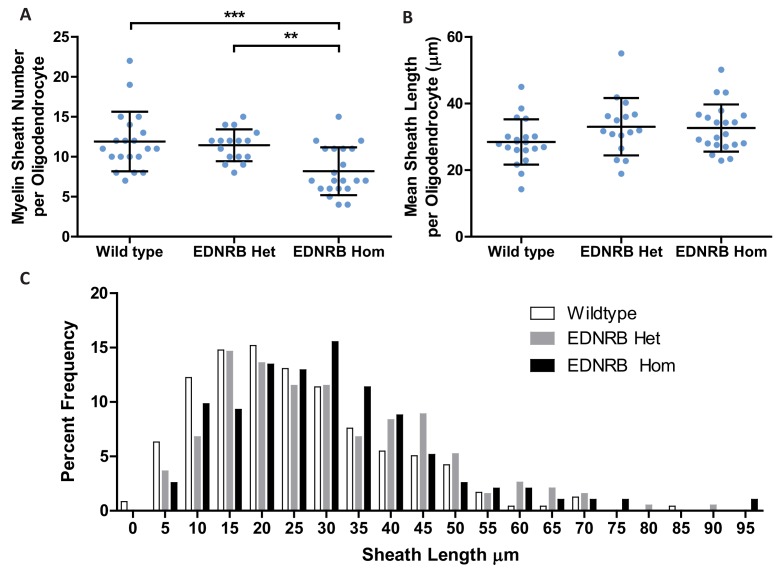
Global loss of EDNRB reduces the number of myelin sheath formed by zebrafish oligodendrocytes. (**A**) Pooled data for number of myelin sheaths formed by zebrafish oligodendrocytes. Wild Type 11.9 ± 3.726 n = 20 cells, EDNRB Het 11.44 ± 1.999 n = 16 cells, EDNRB Hom 8.19 ± 2.994 n = 21 cells from four experiments, (mean ± standard deviation). 1-way ANOVA. (**B**) Pooled data for mean myelin sheath length formed by zebrafish oligodendrocytes. Wild Type 28.45 ± 6.8 n = 20 cells, EDNRB Het 33.04 ± 8.629 n = 16 cells, EDNRB Hom 32.65 ± 7.098 n = 21 from four experiments, (mean ± standard deviation). 1-way ANOVA. (**C**) Frequency distribution of myelin sheath length.

**Figure 5—figure supplement 3.**
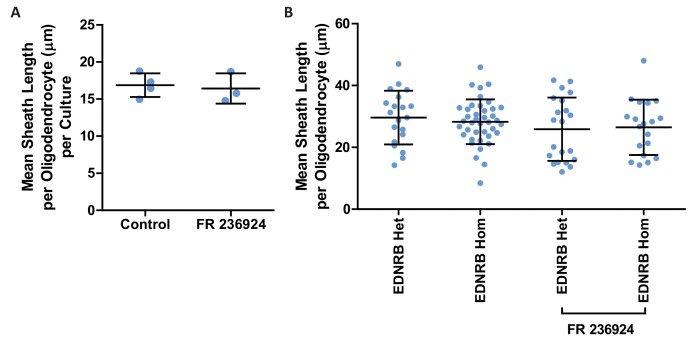
Protein kinase C epsilon activation does not affect myelin sheath length. (**A**) Mean myelin sheath lengths formed by oligodendrocytes in microfiber cultures. Control 16.87 µm ± 0.7932, FR 16.42 µm ± 1.176 n = 3–4 (mean ± standard error). (**B**) Pooled data for number myelin sheath length formed by zebrafish oligodendrocytes. EDNRB Het 29.62 ± 8.685 n = 20 cells, EDNRB Hom 28.27 ± 7.242 n = 41 from, EDNRB Het + FR 236924 25.86 ± 10.25n = 21 cells, EDNRB Hom + FR 236924 26.48 ± 8.951 n = 19 cells (mean ± standard deviation).

### Increasing EDNRB signalling rescues the reduction in myelin sheath number resulting from social isolation

**Figure 6.**
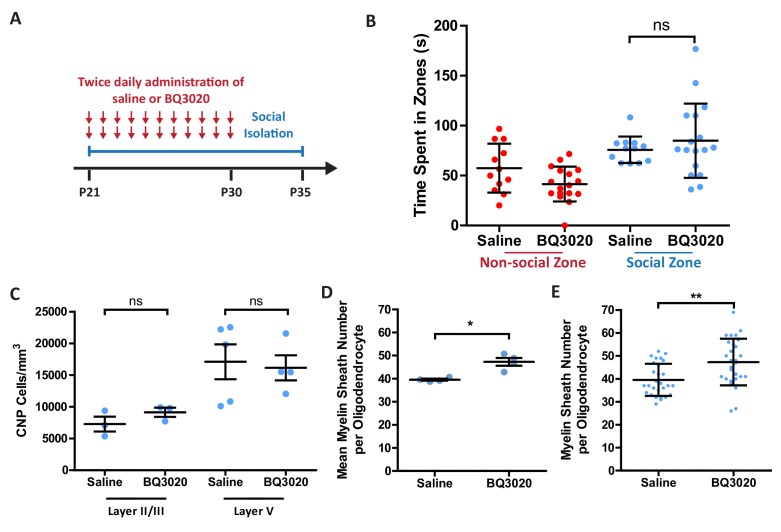
Intranasal administration of an EDNRB agonist rescues the myelin sheath number reduction caused by social isolation. (**A**) Timeline for the intranasal experiment. At postnatal day 21 male mice were housed on their own in isolation. Mice were given two daily administrations of saline or EDNRB agonist BQ3020 from P21-P30. Mice were analysed at P35. (**B**) Time spent within 2.5 cm of non-social container: Saline 57.42 s ± 24.44 n = 12, BQ3020 41.44 ± 17.43 n = 17 and social container: Saline 75.82 s ± 13.26 n = 12, BQ3020 84.96 s ± 37.20 n = 17 (mean ± standard deviation). T-test. (**C**) Quantification of CNP positive cells in medial prefrontal cortex layers II/III: Saline 7278 ± 1165 n = 3 mice, BQ3020 9142 ± 713.3 n = 3 mice (mean ± standard error) and layer V: Saline 17114 ± 2750 n = 5 mice, BQ3020 16154 ± 1980 n = 4 mice (mean ± standard error). (**D**) Mean number of myelin sheath formed by oligodendrocytes per mouse. Saline 39.54 ±0.4301 n = 4 mice, BQ3020 47.29 ± 1.687 n = 4 mice (mean ± standard error). Mann-Whitney test, p=0.0286. (**E**) Pooled data for number of myelin sheaths formed by layer II/III medial prefrontal cortex oligodendrocytes. Saline 39.54 ±6.973 n = 28 cells from four mice, BQ3020 47.29 ± 10.18 n = 28 cells from seven mice (mean ± standard deviation). Mann-Whitney test, p=0.0019.

Our results have shown that social isolation decreases the levels of EDN expression in the CNS vasculature, and that EDNRB signalling in oligodendrocytes influences myelin sheath number. It follows that the decreased EDN signalling is likely contributing to the reduction in myelin sheath numbers seen in the medial prefrontal cortex following social isolation. If so, then we would predict that activation of EDNRB in vivo in the prefrontal cortex would rescue the myelin sheath phenotype associated with social isolation. To test this we used intranasal administration, a technique shown to enable the delivery of peptides into the CNS (Scafidi et al., 2014; Crowe et al., 2018), to introduce the EDNRB agonist BQ3020. Socially isolated mice were given 1 µg BQ3020 twice daily intranasal administration or saline for a period of 10 days from postnatal day 21 to 30 (during the period of isolation), and perfused at postnatal day 35 for analysis of mPFC myelination as above. Importantly, we confirmed that the daily handling involved in this protocol did not negate the effects of social isolation by showing that the sheath numbers in the control isolated mice administered saline had the same reduction in sheath numbers as completely isolated animals (Figure 6B and D–E). Strikingly, and in keeping with our prediction, 10 days of BQ3020 treatment to socially-isolated animals rescued the myelination phenotype normally seen in these mice, with a 20% increase in the number of myelin sheaths formed by mPFC oligodendrocytes in treated animals, meaning that the sheath numbers were now not significantly different to wild-type animals housed in groups (Figure 6D–E).

**Figure 6—figure supplement 1.**
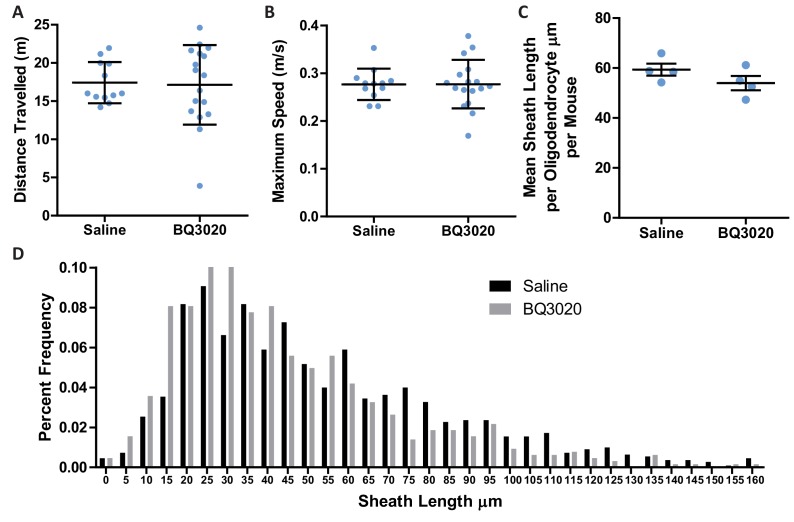
Intranasal administration of EDNRB agonist BQ3020 does not affect myelin sheath length. (**A**) Distance travelled by mice during 5 minutes of exploration of the social interaction assay. Saline 17.48 meters ± 2.697 n = 12 mice, BQ3020 17.13 meters ± 35.209 n = 17 mice (mean ± standard deviation). (**B**) Maximum speed travelled by mice during 5 min of exploration of the social interaction assay. Saline 0.2769 ± 0.03276 n = 12 mice, BQ3020 0.2773 meters ± 0.05085 n = 17 mice (mean ± standard deviation). (**C**) Mean myelin sheath length formed by oligodendrocytes per mouse. Saline 59.38 µm ± 4.831 n = 28 cells from four mice, BQ3020 53.98 µm ± 5.748 n = 28 cells from four mice (mean ± standard deviation). (**D**) Frequency distribution of myelin sheath lengths.

**Figure 6—figure supplement 2.**
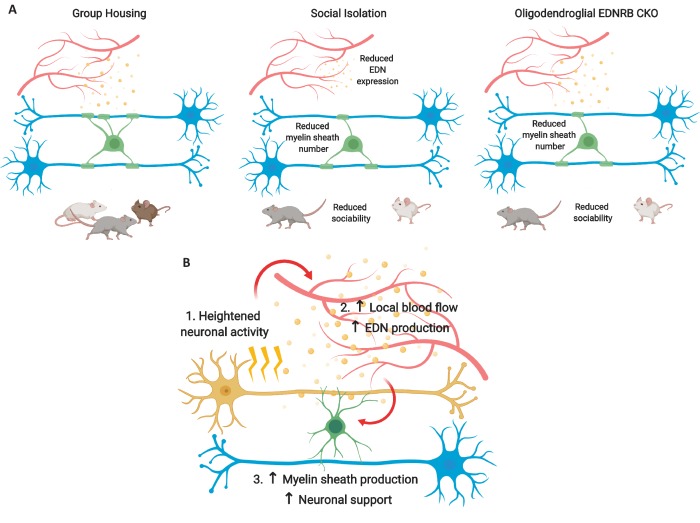
Proposed model for how EDNRB regulates myelin sheath number. (**A**) Summary of results from paper. In a social environment, EDN from the vasculature increases myelin sheath number in the medial prefrontal cortex, influencing sociability. In social isolation vascular EDN production is reduced, leading to a decreased number of myelin sheaths formed by oligodendrocytes and reduced sociability. Oligodendroglial knock out of EDNRB reduces the number of myelin sheaths formed by oligodendrocytes and reduced sociability. (**B**) Proposed hypothesis for how EDN signalling links neuronal activity to increased myelination and thus support of active axons. 1. Increased local neuronal activity signals to the vasculature to increase blood flow to the active area 2. The increased blood flow stimulates EDN production from endothelial cells 3. Increased EDN levels leads to the formation of more myelin sheaths by oligodendrocytes. Figures created with Biorender.

## Discussion

Here we identify a novel signalling pathway in which the oligodendroglial G-protein coupled receptor EDNRB regulates myelin sheath number and the response of oligodendrocytes to social experience (Figure 6—figure supplement 2A). We studied mice following a previously established experimental manipulation in which early postnatal social isolation leads to a reduction in the number of myelin sheaths formed by individual oligodendrocytes in the medial prefrontal cortex (mPFC) of mice. We showed that isolated animals have reduced EDN mRNA expression in the blood vessels of the mPFC. We found that perturbing endothelin signalling in oligodendroglia by a conditional knockout of the EDN receptor EDNRB phenocopies the reduced number of sheaths seen in the isolated animals. In turn intranasal administration of an EDNRB agonist during the period of isolation rescues the effects of social deprivation on myelination. Together with additional experiments in cell culture and zebrafish, which identified the protein kinase C (PKC) epsilon isoform as being activated downstream of EDNRB to regulate myelination by oligodendrocytes, our loss- and gain-of-function experiments demonstrate a role for endothelin signalling in regulating the number of myelin sheaths formed by individual oligodendrocytes.

Our work provides evidence for the importance of the interaction between the vasculature and oligodendroglia during myelination per se, extending observations indicating that the vasculature can influence earlier stages of the oligodendrocyte lineage. For example, during development, oligodendrocyte progenitor cells use blood vessels as a scaffold to migrate along (Tsai et al., 2016). Once in place, local oxygen levels influence their differentiation into oligodendrocytes, stalling the process when levels are low. Only when an adequate oxygen supply is established are the cells able to differentiate and begin the process of myelination (Yuen et al., 2014). Here, we demonstrate a third role – the regulation of myelination itself through the control by EDN of myelin sheath number by individual differentiated oligodendrocytes. Such a role is potentially important, as it provides an indirect mechanism by which the regulation of myelination in the CNS might be linked to levels of neuronal activity. A key function of myelin is the provision of metabolic substrates to the underlying axon via specific transporters within the inner layer of the sheath (Fünfschilling et al., 2012; Lee et al., 2012b; Meyer et al., 2018). These substrates drive ATP production within the axon, so providing a source of energy at some distance from the cell body as required to sustain axonal activity. Prior work has implicated that NMDA receptor activation in oligodendrocytes following glutamate release by active axons in relaying the need for metabolic support of axons to oligodendrocytes (Saab et al., 2016). We now propose that an increase in EDN production by endothelial cells, as predicted to occur in the presence of increased blood flow or hypoxia, will increase myelin sheath generation by local oligodendrocytes and so provide a further means of linking energy supply and demand. Changes in blood flow and hypoxaemia are associated with increased cortical activity (forming the basis of the BOLD signal detected by fMRI). Therefore, an EDN-mediated pathway driving an increase in myelination is a plausible mechanism for ensuring that the metabolic demands of active axons are met. Further work examining the effect of EDNRB loss in oligodendrocytes on axonal function and viability in ageing are required to test this further.

A second important implication of our study relates to the emerging concept of adaptive myelination. This concept argues that myelination in the CNS is plastic, and regulated by differing codes of activity, each eliciting different effects on myelination that in turn alter circuit function. This hypothesis therefore proposes that adaptive myelination represents a form of neural plasticity that could, like synaptic plasticity, enable the brain to modify circuit function in response to experience-in other words, to adapt. However, this hypothesis remains to be fully explored, and a key requirement for these experiments is the identification of the mechanisms that link changes in activity patterns following experience to the changes in oligodendrocyte number and/or sheath formation that could alter circuit function. Prior work has identified a direct role for axon-derived signals in regulating myelination. We now identify a mechanism by which the effects of activity could lead to an increased number of myelin sheaths through indirect signalling via the vasculature. Our findings showing that social isolation reduces endothelial EDN expression, that an oligodendroglial-specific deletion of the relevant EDNRB receptor phenocopies the myelination defect caused by social isolation, and that an EDNRB agonist rescues the myelination defect of individual oligodendrocytes in the mPFC together argue strongly for a role of vascular EDN synthesis in mediating such indirect effects. Our results do not however imply that changes in EDN signalling are solely responsible for the effects of social isolation on myelination. It is clear, for example, that social isolation can affect oligodendrocyte number, a feature that does not appear to be controlled by EDN in the healthy nervous system. Other pathways could contribute to this aspect of adaptive myelination. Such potential pathways are BDNF signalling via oligodendroglia TrkB (Geraghty et al., 2019; Gibson et al., 2019) and glutaminergic signalling via AMPAR-mediated effects on newly differentiating oligodendrocytes (Kougioumtzidou et al., 2017). Further work manipulating EDN expression specifically in endothelial cells during social isolation is required to explore this.

Based on recent live imaging studies showing that myelin sheath numbers in cortical oligodendrocytes remain largely stable throughout life (Hill et al., 2018; Hughes et al., 2018), we propose that any endothelin-mediated regulation of sheath number is likely to occur around the birth of each newly formed oligodendrocyte. The continuous generation of oligodendrocytes in the adult CNS, itself increased by activity-related signals ensures that dynamic regulation of endothelin signalling from the vasculature has the capacity to play a role in adaptive myelination throughout life.

How though might new myelin sheaths formed by individual oligodendrocytes in response to EDN signalling enhance circuit function the cortex? By linking metabolic demand to metabolic support for axons in regions of high activity (as discussed above), EDN signalling in cortical oligodendrocytes could play a central role in enhancing the ability of the axon to sustain higher levels of energy-requiring conduction, so enabling changes in circuit function. An intriguing possibility suggested by a comparison of gene expression in cortical oligodendroglia and spinal cord oligodendroglia showing higher levels of expression of EDNRB in the former (Horiuchi et al., 2017; Marques et al., 2018), is that these cortical oligodendrocytes are specialised for this role linking metabolic demand to support. By contrast, oligodendrocytes in white matter might be more specialised for rapid axonal conduction. Further work examining oligodendrocyte heterogeneity between grey matter and white matter regions of the CNS will be required to test this. Another way in which additional myelin sheaths might impact circuit function is the addition of sheaths to axons in the cortex that are discontinuously and sparsely myelinated (Tomassy et al., 2014; Hill et al., 2018; Hughes et al., 2018). The resulting effects on conduction velocity resulting from short stretches of axons now supporting rapid saltatory conduction could, as has been suggested elsewhere, enable activity-dependent myelination to have a role in signal synchrony (Seidl et al., 2010; Seidl et al., 2014; Freeman et al., 2015; Baraban et al., 2016; Timmler and Simons, 2019). However, such a model requires a very precise link between axonal selection and myelination, and the precision required may be difficult to achieve with a diffusible vascular-derived signal.

Our findings on the role of EDN in myelination complement other investigations demonstrating a role of astrocytic EDN hindering oligodendrocyte differentiation during remyelination (Hammond et al., 2014; Hammond et al., 2015). In agreement with this observation, our global EDNRB mutant zebrafish were found to have an increased number of oligodendrocytes (Figure 5—figure supplement 1). Together previous studies and ours suggest that EDN signalling plays two roles, indirectly inhibiting oligodendrocyte formation, but, promoting myelin sheath formation once differentiation has occurred. At apparent odds with this conclusion, conditional loss of EDNRB from oligodendrocyte progenitors had no effect on remyelination; having deleted OPC EDNRB from adult mice 3 days prior to demyelination of the external capsule, Hammond and colleagues observed that the percentage of remyelinated axons, assessed by electron microscopy, was unchanged 14 days after injury (Hammond et al., 2015). However, this divergence may result from the different microenvironments of developmental myelination and remyelination or from intrinsic differences between cortical and white matter oligodendrocytes, as discussed above, and further studies examining grey matter remyelination are required to resolve the point.

The conclusion that EDN has contrasting effects on oligodendrocyte formation and on myelination highlights that these two steps in oligodendrocyte development are regulated independently. This in turn has important implications for the development of regenerative therapies for the progressive neurodegenerative phase of the demyelinating disease Multiple Sclerosis (MS). Current strategies seeking to re-purpose FDA approved drugs to enhance remyelination, and so restore the neuroprotective effect of the myelin sheath to the axon, have focused largely on the differentiation step from precursor cell to oligodendrocyte, with one screen using micropillar arrays to examine the later stage of wrapping (Mei et al., 2014). None however specifically examine the key final step-formation of the sheath itself. Neuropathological studies of MS lesions revealing pre-myelinating oligodendrocytes unable to complete remyelination suggest for that failure of this final step may contribute to pathology. These findings emphasise that the already differentiated oligodendrocyte represents a possible target for remyelination strategies in MS, a conclusion reinforced by recent papers using Carbon14 dating, electron microscopy or snRNA seq of post mortem human MS material providing evidence that pre-existing adult oligodendrocytes contribute to repair (Duncan et al., 2018; Jäkel et al., 2019; Yeung et al., 2019). Strategies to identify targets within pathways such as that activated by EDNRB that promote sheath formation directly will therefore be an important addition to the current approaches being taken in drug discovery for progressive multiple sclerosis.

## Materials and methods

**Key resources table:** 

Reagent type (species) or resource	Designation	Source or reference	Identifiers	Additional information
Genetic reagent (*M. musculus*)	Ednrb flox/flox	The university of Edinburgh, Bagnall et al., 2006; Ge et al., 2006		
Genetic reagent (*M. musculus*)	Pdgfra-cre	Jackson labs	#013148	
Genetic reagent (*D. rerio*)	Rse Tlf802	Frohnhöfer et al., 2013; Krauss et al., 2014		
Genetic reagent (*D. rerio*)	Tg(mbp:EGFP)	The University of Edinburgh, Almeida et al., 2011		
Antibody	Mouse monoclonal anti CNPase	Atlas	AMAb91072	1:2000
Antibody	Rat monoclonal anti MBP	Serotec	MCA409S	1:250
Antibody	Rat monoclonal anti PECAM1	BD Pharmingen	550274	1:100
Antibody	Rabbit monoclonal anti S100β	Thermo	MA5-12969	1:100
Antibody	Rabbit monoclonal anti IBA1	Abcam	ab178846	1:500
Antibody	Rabbit polyclonal anti Laminin	Abcam	ab11575	1:300
Antibody	Rabbit polyclonal anti OLIG2	Millipore	ab9610	1:100
Antibody	Mouse monoclonal anti CC1	Abcam	ab16794	1:300
Antibody	Mouse monoclonal anti GAPDH	Millipore	MAB374	1:1000
Antibody	Mouse monoclonal anti Beta actin	Abcam	ab 8226	1:1000
Antibody	Rabbit polyclonal anti, Phospho-PKCε S729	Abcam	88241	1:1000
Commercial assay, kit	Phospho-explorer antibody array	Full Moon Biosystems,	Phospho-explorer array (PEX100)	
Sequence-based reagent	*Edn1*	Advanced Cell Diagnostics	435221	
Sequence-based reagent	*Edn2*	Advanced Cell Diagnostics	418221	
Sequence-based reagent	*Edn3*	Advanced Cell Diagnostics	Custom made	
Sequence-based reagent	*Pecam1*	Advanced Cell Diagnostics	316721-C3	
Sequence-based reagent	*Olig2*	Advanced Cell Diagnostics	447091	
Sequence-based reagent	*Ednrb*	Advanced Cell Diagnostics	473801	
Peptide, recombinant protein	BQ3020	Tocris	1189	100 ng/mL
Chemical compound, drug	BQ788	Tocris	1500	100 ng/mL
Chemical compound, drug	FR236924	Tocris	0373	25 µM
Other	Microfibers	The Electrospinning company		1–2 mcro diameter poly-l-lactic acid
Software, algorithm	Any-maze software	http://www.anymaze.co.uk/		
Software, algorithm	ImageJ	https://imagej.nih.gov/ij/		
Software, algorithm	Graphpad Prism	https://www.graphpad.com/scientific-software/prism/		

### Mice

Animal husbandry and experiments were performed under UK Home Office project licenses issued under the Animals (Scientific Procedures) Act. *Ednrb*^flox/flox^ (Bagnall et al., 2006; Ge et al., 2006) were generously provided by Professor David Webb and Professor Yuri Kotelevtsev (Edinburgh University) where exons 3 and 4 are flanked with Cre-LoxP sites. Homozygous mice for the EDNRB floxed allele were crossed to *Pdgfra*-cre mice obtained from Jackson laboratories (013148). Offspring were then backcrossed to create mice Heterozygous for the floxed allele and carriers for the Cre transgene. Experimental mice were obtained by crossing animals heterozygous for the floxed *Ednrb* allele and carriers of the Cre transgene generating both *Ednrb* wild type control and *Ednrb* floxed homozygous mice in the same litters. Mice were genotyped by transnetyx and confirmed as a CKO by performing RNAScope for *Ednrb* (described below) Mice of each genotype were used at the ages and in the numbers stated in the Results. For the social isolation experiments, postnatal day 21 male mice were either group housed in a regular social environment, containing 3–5 mice, or in isolation, for 2 weeks.

### Sociability test

At postnatal day 35 mice were allowed to freely explore an open field arena for 5 min containing two identical wire mesh containers. One container housed an unrelated male wild type mouse of a similar age, while the other remained empty. Mice were allowed to explore the arena for 5 min and the duration of time during which the mouse of interest came within 2.5 cm of either container was automatically recorded using Any-maze software. Speed and distance travelled were also recorded by the software. In all tests, mice were assessed with the experimenter blind to genotype.

### Intranasal administration

Mice were given brief isoflurane inhalation anaesthesia before being held upside down. Using a pipette, 5 μL of either saline or 1 µg BQ3020 (Tocris - 1189) in saline was placed on either nostril. This was repeated twice daily (9am and 5pm) from postnatal day 21 to postnatal day 31.

### Antibodies

CNPase Atlas AMAb91072 (1:2000), MBP Serotec MCA409S (1:250), PECAM1 BD Pharmingen 550274 (1:100), S100ß Thermo MA5-12969 (1:100), IBA1 Abcam ab178846 (1:500), Laminin Abcam ab11575 (1:300), Olig2 Millipore AB9610 (1:100), CC1 Abcam ab16794 (1:300).

### Immunofluorescence staining - Cryosections

Animals were intracardially perfused with 4% PFA (wt/vol; Sigma) in PBS, after which brains were post-fixed overnight and then cryoprotected in sucrose prior to embedding in OCT and storage at −80°C. Brains were cryosectioned coronally at a thickness of 16 μm using a Thermo cryostat and mounted onto Superfrost Plus slides. Sections were blocked for 1 hr at room temperature in 10% goat serum, 0.1% triton in PBS. Primary antibodies were incubated in block solution at 4°C overnight. Sections were washed with PBS for 3 × 15 min at room temperature and stained using species-specific Alexa fluorophore-conjugated antibodies in block solution for 1 hr at room temperature. Sections were washed in PBS for a further 3 × 15 min and stained with Hoechst for 5 min and mounted onto slides with fluoromount G. Mouse medial prefrontal cortex was defined as the infralimbic and prelimbic areas between bregma 1.7 mm and 2 mm and visual cortex bregma −3.5 mm and −4.5 mm.

### Immunofluorescence staining - Vibratome Sections for Oligodendrocyte morphological analysis

Animals were intracardially perfused with 4% PFA (wt/vol; Sigma) in PBS, after which brains were post-fixed overnight and embedded in 2% low melting point agarose. Using a Leica vibratome, 100 µM coronal free-floating sections were cut. Sections underwent antigen retrieval in 0.05% Tween20, 10 mM tri-sodium citrate (pH 6.0) at 95°C for 20 min. Sections were then blocked for 3 hr at room temperature in 10% goat serum, 0.25% triton in PBS. Primary antibodies were incubated in block solution at 4°C on a rocker for 24 hr. Sections were washed with PBS for 3 hr at room temperature and stained using species-specific Alexa fluorophore-conjugated antibodies in block solution for 4 hr at room temperature. Sections were washed in PBS for a further 3 hr and stained with Hoechst for 20 min and mounted onto slides with fluoromount G. Sections were analysed with the experimenter blind to experimental condition and/or genotype as below.

To analyse oligodendrocyte number 10 fields of 40x magnification were taken from 2 100 µm sections per mouse of cortical layer II/III or layer V using a SP8 confocal microscope. CNP positive, Olig2 and CC1 cells were counted.

To analyse oligodendrocyte morphology, random areas of the medial prefrontal cortex layer II/III were imaged at 63x magnification using an SP8 confocal microscope. seven individual CNP positive oligodendrocytes per mouse, each with all myelin sheaths present within the 100 µm section as assessed by following each process from the cell body and ensuring none exited the section, were imaged using a z step size of 0.5 µm. Analysis was performed using an ImageJ plugin – simple neurite tracer (Longair et al., 2011).

### RNAScope In situ hybridisation

Cryosections (cut as above) were processed as recommended by Advanced Cell Diagnostics. Briefly sections were dried overnight at 60°C, after which they were incubated at 40°C with pre-treatment 4 for 30 min before incubation with RNAScope probes for 2 hr at 40°C. RNAScope probes used were; *Edn1* (435221), *Edn2* (418221), *Edn3* (custom made), *Pecam1* (316721-C3), *Oolig2* (447091-C2), *Ednrb* (473801). Following the RNAScope protocol, sections were stained as above.

For analysis, 5 random 63x areas of medial prefrontal cortex layer II/III were imaged using a SP8 confocal microscope. Cells positive for *Pecam1* and *Edn1/3* were counted. Sections were analysed with the experimenter blind to experimental condition and/or genotype.

### Rat oligodendrocyte precursor cell culture

Rat OPCs were prepared from mixed glial cultures as described previously (McCarthy, 1980; Bechler et al., 2015). Briefly, cortices of postnatal day 0–2 Sprague Dawley rats were dissected out. The tissue was digested with 1.2 Units/mL papain, 0.1 mg/mL L-cysteine and 0.40 mg/mL DNase for 1 hr at 37°C. Tissue was cultured in DMEM, 10% FCS, 1% P/S in T75 flasks, pre-coated with 5 µg/mL poly-D-lysine, at a density of 1.5 brains per flask. Cells were grown at 37°C in 7.5% CO_2_ with medium changes every 2–3 days. After 10–12 days cells were mechanically separated on an orbital shaker at 250 rpm, 37°C. Loosely attached microglia were removed by shaking for 1 hr. Further shaking for 16–18 hr detached OPCs. Cell yield was counted using a haemocytometer and plated in assay-dependent conditions as below.

### Microfiber cultures

Custom parallel-aligned microfibers were purchased from the Electrospinning Company. 1–2 micron diameter poly-l-lactic acid microfibers were synthesised and suspended over plastic scaffolds fitting into 12-well tissue culture plates. Microfibers were washed with 70% EtOH for 10 min followed by coating with PDL for 1 hr at 37°C in a 12 well tissue culture plate. Microfibers were washed twice with sterile water and left in preheated myelination media. 35,000 rat OPCs and 50,000 mouse OPCs in myelination media (50:50 DMEM:Neurobasal Media, B27 (Invitrogen), 5 µg/mL N-acetyl cysteine, and 10 ng/mL D-biotin, ITS, and modified Sato (100 µg/mL BSA fraction V, 60 ng/ml Progesterone, 16 µg/ml Putrecsine, 400 ng/mL Tri-iodothyroxine, 400 ng/mL L-Thyroxine; reagents from Sigma-Aldrich)) were triturated to break up cell clumps and added dropwise to the microfibers. Cells were left to recover for 3 days before media changing and addition of treatment, followed by subsequent media changes every 3 days.

After 14 days of culture cells were fixed in 4% PFA for 15 min. To visualise myelination, cells were permeabilised with 0.1% Triton-X for 10 min and stained with for MBP (1:250) overnight at 4°C followed by incubation with Alexa 488-conjugated goat ant-rat (1:1000) for 1 hr at room temperature and Hoescht for 5 min to visualise nuclei.

To analyse myelination 15–30 individual myelinating oligodendrocytes from one coverslip were imaged (with the experimenter blind to condition) using an SP8 confocal at 40x magnification with a z-step of 0.35 µm. The same settings (laser power, gain, offset etc.) were used between coverslips. ImageJ was used to analyse myelination, again with the experimenter blind to condition. A sheath was defined as a continuous MBP positive wrap fully surrounding a microfiber as assessed using the 0.35 µm z-series. Concentric tubes were traced and the length measured. In addition the number of concentric sheaths made per individual oligodendrocyte was recorded. Sheath lengths were grouped into 5 µm bins and the frequency from one experiment calculated. Mean frequencies from at least three experiments were generated and plotted as a frequency distribution.

Drugs used: BQ3020 (100 ng/mL, Tocris - 1189) in 0.03M sodium bicarbonate, BQ788 (100 ng/mL, Tocris - 1500) in DMSO, FR236924 (25 µM, Tocris - 0373) in DMSO.

### Mouse oligodendrocyte precursor cell culture

Mouse OPCs were isolated from P6-P9 pups as described (Watkins et al., 2008). Ear clips were taken for subsequent genotyping. Briefly, cerebral cortices were dissected, diced and dissociated into single-cell suspensions gently using MACS Neural Tissue Dissociation Kit P (130-092-628). Cells were resuspended in 0.2% BSA, Insulin, PBS and transferred to treated tissue culture dishes coated with BSL1 (L-1100, Vector Labs) for 15 min twice. Cell solutions were then transferred to dishes coated with anti-PDGFRα (CD140a) for 45 min. Solutions were aspirated and attached cells washed twice with media and removed with a cell scraper. All collected cells were added to vented T75 flasks and grown at 37°C 7.5 CO_2_. Cells were grown in myelination media containing PDGF and NT3 and changed every 2 days and supplemented daily with PDGF. After 7–9 days confluent flasks were washed with PBS and then detached using TrypLE for 10 min at 37°C. Solutions were centrifuged at 1000 rpm for 5 min, resuspended and counted using a haemocytometer.

### qPCR

75,000 mouse OPCs were cultured on pre-coated PDL six well plates for 2 days. RNA was extracted from cells using a Qiagen RNeasy mini kit. RNA was then converted to cDNA libraries using a SuperScript First-Strand Synthesis System. Sybr green qPCR was performed using primers either bought from Qiagen or designed at 0.5 µM. qPCR was performed on a LightCycler 480 II. CT values from designed primers were normalised against GAPDH purchased from Qiagen.

### Full moon Phospho-Explorer antibody array

Processing of the phospho-explorer array (PEX100) was performed following the Full Moon Biosystems guidelines. OPCs were immunopanned from wildtype mice and expanded in the presence of PDGF. Upon confluency, OPCs were differentiated through supplementation of CNTF, NT3 and T3. After 2 days of differentiation cells were starved for 4–5 hr in media devoid of supplementation to remove stimulation from the media and then treated for 15 min with either vehicle or BQ3020 (100 ng/mL). Cells were lysed in RIPA buffer containing protease and phosphatase inhibitors (Calbiochem – 539134 and 524621) and processed for the array as recommended by Full Moon Biosystems.

Briefly, proteins present in the lysates were biotinylated through incubation with biotin. Chips were blocked using milk and biotinylated protein lysates were washed over. Binding of the individual proteins to each antibody spot on the array was assessed through fluorescent labelling with a dye-labelled streptavidin read using an Innopsys 710-IR scanner and analysed using Mapix software.

For each antibody the background intensity was subtracted, dye signal normalised and an average calculated of the duplicate spots. The ratio was calculated of binding to the phosphorylated amino acids vs the binding to the non-modified regions of the protein for each molecule, calculating this for both control and BQ3020 treated cells. The fold change in phosphorylation for each targeted amino acid was generated by comparing BQ3020 to vehicle. For selection a fold change of greater than two and less than 0.5 was set as the cut-off.

Antibody array was performed once – one cell lysate per condition.

### Western blot

For western blot analysis, 9 cm treated plastic tissue culture dishes were coated with PDL for either 1 hr at 37°C or overnight at room temperature. 1 million OPCs were added in myelination media to coated plates. After 3 days in culture, oligodendrocytes were starved from media supplementation through incubation with DMEM and 1% P/S only for 4–5 hr. Fresh starvation media was then added containing either vehicle control, BQ3020 (100 ng/mL) or FR236924 (25 µM). After 10 minutes cells were lysed and scraped into RIPA buffer with protease and phosphatase inhibitors (Calbiochem – 539134 and 524621) for 10 min on ice. Lysates were spun at 16,000 g for 10 min and supernatants retained.

Protein concentration was estimated using a BCA assay kit and loaded into precast protein gels with a protein marker of known molecular weights. Gels were ran at 60 volts for 30 min and then increased to 100 volts for 1 hr. Protein was transferred from the gels to nitrocellulose membranes pre-treated with methanol at 400 mA for 2 hr on ice. Membranes were blocked in 4% BSA in TBS- 0.1% Tween for 40 min and incubated with primary antibodies (GAPDH Millipore MAB374, Beta-actin Abcam ab8226, Phospho-PKCε S729 Abcam 88241) overnight at 4°C on an orbital shaker. Membranes were washed in TBS-Tween for 30 min and incubated with species specific secondary horseradish peroxide antibodies at room temperature for 1 hr. Membranes were washed for a further 30 min and incubated with ECL2 for 5 min. Blots were detected using a Licor scanner. Subsequent western blots were performed using the same membranes following removal of bound antibodies through incubation with stripping buffer for 15 min.

### Zebrafish

Animal husbandry and experiments were maintained in accordance with UK Home Office guideline. The following zebrafish lines were used: Wild type WIK and AB, Tg(mbp:EGFP) and EDNRB Hom (Rse tLF802) (Frohnhöfer et al., 2013; Krauss et al., 2014). Rse zebrafish contain a point mutation in the *ednrba* gene at amino acid 163 where glutamine is substituted for lysine. This mutation creates a restriction site for the enzyme PsiI. Touchdown PCR was performed using a light cycler. To distinguish between wild type and homozygous mutants, the PCR product was incubated with restriction enzyme PsiI for 3 hr at 37°C and run on a 2% agarose TAE gel. Wild type DNA remains uncut at 363 bp whereas homozygous DNA generates two similar sized bands of 187 and 176 bp.

Rse Homozygous mutants were crossed to the stable transgenic line Tg(mbp:EGFP) expressing EGFP in all oligodendrocytes previously generated in the Lyons lab (Almeida et al., 2011). Five dpf larval zebrafish were embedded in 1.3% low melting point agarose with tricane. Lateral images of zebrafish spinal cords were taken on an Apatome2 microscope at 20x and stitched together using ZEN imaging software. Using ImageJ cell counter, GFP fluorescent oligodendrocytes were counted along the entire length of the dorsal and ventral spinal cord. Fish were genotyped after imaging and analysis – experimenters were therefore blinded to genotype during the data acquisition.

Offspring of the paired mating of either Rse heterozygotes or Rse heterozygotes to Rse homozygotes were subject to single oligodendrocyte analysis. Single oligodendrocytes were labelled using the mbp:EGFP-CAAX plasmid developed within the Lyons lab (Almeida et al., 2011). In brief, plasmid DNA generated above for the mbp:EGFP transgenic line was adapted through the addition of the four amino acid CAAX motif therefore anchoring GFP to membranes of oligodendrocytes and myelin sheaths. Mosaic fish were generated by injecting fertilised eggs with 1 nL of a solution of 12.5 ng/µL mbp:EGFP-CAAX plasmid DNA and 12.5 ng/µL tol2 transposase mRNA between the 1–8 cell stage. Transposase mRNA was produced using an Ambion message machine kit SP6 from pre-digested DNA. Injections were performed using pulled glass needles using gas to force out a precise volume, measured prior to injection in a drop of mineral oil on a calibration slide. four dpf larval zebrafish were screened for fluorescent cells and embedded in 1.3% low melting point agarose with tricane. Lateral images of individual cells were taken on a Zeiss LSM710 confocal. Analysis was performed using ImageJ. GFP fluorescent sheaths longer than 5 µm were traced and the length measured. The number of sheaths made by individual oligodendrocytes was recorded and the mean sheath length was calculated per oligodendrocyte. Fish were genotyped after imaging and analysis - experimenters were therefore blinded to genotype during the data acquisition.

For the experiments asking whether a protein kinase C agonist would rescue myelination defects in the Rse Homozygous mutants, injected fish were treated with either 1%DMSO or 10 μM FR236924 in DMSO at 3 dpf for 24 hr before image acquisition.

### Statistics

All analysis of cell counts and myelination and were performed on ImageJ blind to condition using a filename randomiser macro. Data is presented showing standard deviations to show the variability, or standard error of the mean when averaged data calculated as a mean per animal or per culture was used for each n. Statistical analysis was performed using GraphPad Prism software. Data was tested for normality using a Kolmogorov–Smirnov test. When data fitted a normal distribution parametric t-tests and 1-way ANOVA, with Tukey’s post hoc, were used. Where data did not meet normal distribution non-parametric Mann-Whitney U tests and Kruskal-Wallis tests, with Dunns post hoc, were used.

## Data Availability

Data generated from phosphorylation screen is included in Supplementary file 1.

## Additional files

Supplementary file 1. Antibody array of phosphorylation events downstream of EDNRB. Wild type mouse oligodendrocytes were starved for 4–5 hr in media devoid of supplementation and then treated for 15 min with either vehicle or BQ3020 (100 ng/mL). For each antibody the background intensity was subtracted, dye signal normalised and an average calculated of the duplicate spots. The ratio was calculated of binding to the phosphorylated amino acids vs the binding to the non-modified regions of the protein for each molecule, calculating this for both control and BQ3020 treated cells. The fold change in phosphorylation for each targeted amino acid was generated by comparing BQ3020 to vehicle. For selection a fold change of greater than 2 and less than 0.5 was set as the cut-off. Antibody array was performed once – one cell lysate per condition.

Transparent reporting form

## References

[bib1] Almeida RG, Czopka T, Ffrench-Constant C, Lyons DA (2011). Individual axons regulate the myelinating potential of single oligodendrocytes in vivo. Development.

[bib2] Bagnall AJ, Kelland NF, Gulliver-Sloan F, Davenport AP, Gray GA, Yanagisawa M, Webb DJ, Kotelevtsev YV (2006). Deletion of endothelial cell endothelin B receptors does not affect blood pressure or sensitivity to salt. Hypertension.

[bib3] Baraban M, Mensch S, Lyons DA (2016). Adaptive myelination from fish to man. Brain Research.

[bib4] Bechler ME, Byrne L, Ffrench-Constant C (2015). CNS myelin sheath lengths are an intrinsic property of oligodendrocytes. Current Biology.

[bib5] Crowe TP, Greenlee MHW, Kanthasamy AG, Hsu WH (2018). Mechanism of intranasal drug delivery directly to the brain. Life Sciences.

[bib6] Dancu MB, Berardi DE, Vanden Heuvel JP, Tarbell JM (2004). Asynchronous shear stress and circumferential strain reduces endothelial NO synthase and cyclooxygenase-2 but induces endothelin-1 gene expression in endothelial cells. Arteriosclerosis, Thrombosis, and Vascular Biology.

[bib7] Duncan ID, Radcliff AB, Heidari M, Kidd G, August BK, Wierenga LA (2018). The adult oligodendrocyte can participate in remyelination. PNAS.

[bib8] Etxeberria A, Hokanson KC, Dao DQ, Mayoral SR, Mei F, Redmond SA, Ullian EM, Chan JR (2016). Dynamic modulation of myelination in response to visual stimuli alters optic nerve conduction velocity. Journal of Neuroscience.

[bib9] Foster AY, Bujalka H, Emery B (2019). Axoglial interactions in myelin plasticity: evaluating the relationship between neuronal activity and oligodendrocyte dynamics. Glia.

[bib10] Freeman SA, Desmazières A, Simonnet J, Gatta M, Pfeiffer F, Aigrot MS, Rappeneau Q, Guerreiro S, Michel PP, Yanagawa Y, Barbin G, Brophy PJ, Fricker D, Lubetzki C, Sol-Foulon N (2015). Acceleration of conduction velocity linked to clustering of nodal components precedes myelination. PNAS.

[bib11] Frohnhöfer HG, Krauss J, Maischein H-M, Nüsslein-Volhard C (2013). Iridophores and their interactions with other chromatophores are required for stripe formation in zebrafish. Development.

[bib12] Fünfschilling U, Supplie LM, Mahad D, Boretius S, Saab AS, Edgar J, Brinkmann BG, Kassmann CM, Tzvetanova ID, Möbius W, Diaz F, Meijer D, Suter U, Hamprecht B, Sereda MW, Moraes CT, Frahm J, Goebbels S, Nave KA (2012). Glycolytic oligodendrocytes maintain myelin and long-term axonal integrity. Nature.

[bib13] Ge Y, Bagnall A, Stricklett PK, Strait K, Webb DJ, Kotelevtsev Y, Kohan DE (2006). Collecting duct-specific knockout of the endothelin B receptor causes hypertension and sodium retention. American Journal of Physiology-Renal Physiology.

[bib14] Geraghty AC, Gibson EM, Ghanem RA, Greene JJ, Ocampo A, Goldstein AK, Ni L, Yang T, Marton RM, Paşca SP, Greenberg ME, Longo FM, Monje M (2019). Loss of adaptive myelination contributes to methotrexate Chemotherapy-Related cognitive impairment. Neuron.

[bib15] Gibson EM, Purger D, Mount CW, Goldstein AK, Lin GL, Wood LS, Inema I, Miller SE, Bieri G, Zuchero JB, Barres BA, Woo PJ, Vogel H, Monje M (2014). Neuronal activity promotes oligodendrogenesis and adaptive myelination in the mammalian brain. Science.

[bib16] Gibson EM, Nagaraja S, Ocampo A, Tam LT, Wood LS, Pallegar PN, Greene JJ, Geraghty AC, Goldstein AK, Ni L, Woo PJ, Barres BA, Liddelow S, Vogel H, Monje M (2019). Methotrexate chemotherapy induces persistent Tri-glial dysregulation that underlies Chemotherapy-Related cognitive impairment. Cell.

[bib17] Hammond TR, Gadea A, Dupree J, Kerninon C, Nait-Oumesmar B, Aguirre A, Gallo V (2014). Astrocyte-derived endothelin-1 inhibits remyelination through notch activation. Neuron.

[bib18] Hammond TR, McEllin B, Morton PD, Raymond M, Dupree J, Gallo V (2015). Endothelin-B receptor activation in astrocytes regulates the rate of oligodendrocyte regeneration during remyelination. Cell Reports.

[bib19] Hill RA, Li AM, Grutzendler J (2018). Lifelong cortical myelin plasticity and age-related degeneration in the live mammalian brain. Nature Neuroscience.

[bib20] Hines JH, Ravanelli AM, Schwindt R, Scott EK, Appel B (2015). Neuronal activity biases axon selection for myelination in vivo. Nature Neuroscience.

[bib21] Horiuchi M, Suzuki-Horiuchi Y, Akiyama T, Itoh A, Pleasure D, Carstens E, Itoh T (2017). Differing intrinsic biological properties between forebrain and spinal oligodendroglial lineage cells. Journal of Neurochemistry.

[bib22] Hughes EG, Orthmann-Murphy JL, Langseth AJ, Bergles DE (2018). Myelin remodeling through experience-dependent oligodendrogenesis in the adult somatosensory cortex. Nature Neuroscience.

[bib23] Ishibashi T, Dakin KA, Stevens B, Lee PR, Kozlov SV, Stewart CL, Fields RD (2006). Astrocytes promote myelination in response to electrical impulses. Neuron.

[bib24] Jäkel S, Agirre E, Mendanha Falcão A, van Bruggen D, Lee KW, Knuesel I, Malhotra D, Ffrench-Constant C, Williams A, Castelo-Branco G (2019). Altered human oligodendrocyte heterogeneity in multiple sclerosis. Nature.

[bib25] Koudelka S, Voas MG, Almeida RG, Baraban M, Soetaert J, Meyer MP, Talbot WS, Lyons DA (2016). Individual neuronal subtypes exhibit diversity in CNS myelination mediated by synaptic vesicle release. Current Biology.

[bib26] Kougioumtzidou E, Shimizu T, Hamilton NB, Tohyama K, Sprengel R, Monyer H, Attwell D, Richardson WD (2017). Signalling through AMPA receptors on oligodendrocyte precursors promotes myelination by enhancing oligodendrocyte survival. eLife.

[bib27] Krauss J, Frohnhöfer HG, Walderich B, Maischein HM, Weiler C, Irion U, Nüsslein-Volhard C (2014). Endothelin signalling in Iridophore development and stripe pattern formation of zebrafish. Biology Open.

[bib28] Lee S, Leach MK, Redmond SA, Chong SY, Mellon SH, Tuck SJ, Feng ZQ, Corey JM, Chan JR (2012a). A culture system to study oligodendrocyte myelination processes using engineered nanofibers. Nature Methods.

[bib29] Lee Y, Morrison BM, Li Y, Lengacher S, Farah MH, Hoffman PN, Liu Y, Tsingalia A, Jin L, Zhang PW, Pellerin L, Magistretti PJ, Rothstein JD (2012b). Oligodendroglia metabolically support axons and contribute to neurodegeneration. Nature.

[bib30] Liu J, Dietz K, DeLoyht JM, Pedre X, Kelkar D, Kaur J, Vialou V, Lobo MK, Dietz DM, Nestler EJ, Dupree J, Casaccia P (2012). Impaired adult myelination in the prefrontal cortex of socially isolated mice. Nature Neuroscience.

[bib31] Longair MH, Baker DA, Armstrong JD (2011). Simple Neurite Tracer: open source software for reconstruction, visualization and analysis of neuronal processes. Bioinformatics.

[bib32] Makinodan M, Rosen KM, Ito S, Corfas G (2012). A critical period for social experience-dependent oligodendrocyte maturation and myelination. Science.

[bib33] Marques S, van Bruggen D, Vanichkina DP, Floriddia EM, Munguba H, Väremo L, Giacomello S, Falcão AM, Meijer M, Björklund ÅK, Hjerling-Leffler J, Taft RJ, Castelo-Branco G (2018). Transcriptional convergence of oligodendrocyte lineage progenitors during development. Developmental Cell.

[bib34] McCarthy KD (1980). Preparation of separate astroglial and oligodendroglial cell cultures from rat cerebral tissue. The Journal of Cell Biology.

[bib35] McKenzie IA, Ohayon D, Li H, de Faria JP, Emery B, Tohyama K, Richardson WD (2014). Motor skill learning requires active central myelination. Science.

[bib36] Mei F, Fancy SPJ, Shen YA, Niu J, Zhao C, Presley B, Miao E, Lee S, Mayoral SR, Redmond SA, Etxeberria A, Xiao L, Franklin RJM, Green A, Hauser SL, Chan JR (2014). Micropillar arrays as a high-throughput screening platform for therapeutics in multiple sclerosis. Nature Medicine.

[bib37] Mensch S, Baraban M, Almeida R, Czopka T, Ausborn J, El Manira A, Lyons DA (2015). Synaptic vesicle release regulates myelin sheath number of individual oligodendrocytes in vivo. Nature Neuroscience.

[bib38] Meyer N, Richter N, Fan Z, Siemonsmeier G, Pivneva T, Jordan P, Steinhäuser C, Semtner M, Nolte C, Kettenmann H (2018). Oligodendrocytes in the Mouse Corpus Callosum Maintain Axonal Function by Delivery of Glucose. Cell Reports.

[bib39] Mitew S, Gobius I, Fenlon LR, McDougall SJ, Hawkes D, Xing YL, Bujalka H, Gundlach AL, Richards LJ, Kilpatrick TJ, Merson TD, Emery B (2018). Pharmacogenetic stimulation of neuronal activity increases myelination in an axon-specific manner. Nature Communications.

[bib40] Pandit MM, Inscho EW, Zhang S, Seki T, Rohatgi R, Gusella L, Kishore B, Kohan DE (2015). Flow regulation of endothelin-1 production in the inner medullary collecting duct. American Journal of Physiology-Renal Physiology.

[bib41] Saab AS, Tzvetavona ID, Trevisiol A, Baltan S, Dibaj P, Kusch K, Möbius W, Goetze B, Jahn HM, Huang W, Steffens H, Schomburg ED, Pérez-Samartín A, Pérez-Cerdá F, Bakhtiari D, Matute C, Löwel S, Griesinger C, Hirrlinger J, Kirchhoff F, Nave K-A (2016). Oligodendroglial NMDA receptors regulate glucose import and axonal energy metabolism. Neuron.

[bib42] Sampaio-Baptista C, Khrapitchev AA, Foxley S, Schlagheck T, Scholz J, Jbabdi S, DeLuca GC, Miller KL, Taylor A, Thomas N, Kleim J, Sibson NR, Bannerman D, Johansen-Berg H (2013). Motor skill learning induces changes in white matter microstructure and myelination. Journal of Neuroscience.

[bib43] Sampaio-Baptista C, Johansen-Berg H (2017). White matter plasticity in the adult brain. Neuron.

[bib44] Scafidi J, Hammond TR, Scafidi S, Ritter J, Jablonska B, Roncal M, Szigeti-Buck K, Coman D, Huang Y, McCarter RJ, Hyder F, Horvath TL, Gallo V (2014). Intranasal epidermal growth factor treatment rescues neonatal brain injury. Nature.

[bib45] Scholz J, Klein MC, Behrens TEJ, Johansen-Berg H (2009). Training induces changes in white-matter architecture. Nature Neuroscience.

[bib46] Seidl AH, Rubel EW, Harris DM (2010). Mechanisms for adjusting interaural time differences to achieve binaural coincidence detection. Journal of Neuroscience.

[bib47] Seidl AH, Rubel EW, Barría A (2014). Differential conduction velocity regulation in ipsilateral and contralateral collaterals innervating brainstem coincidence detector neurons. The Journal of Neuroscience.

[bib48] Suminaite D, Lyons DA, Livesey MR (2019). Myelinated axon physiology and regulation of neural circuit function. Glia.

[bib49] Timmler S, Simons M (2019). Grey matter myelination. Glia.

[bib50] Tomassy GS, Berger DR, Chen HH, Kasthuri N, Hayworth KJ, Vercelli A, Seung HS, Lichtman JW, Arlotta P (2014). Distinct profiles of myelin distribution along single axons of pyramidal neurons in the neocortex. Science.

[bib51] Tsai HH, Niu J, Munji R, Davalos D, Chang J, Zhang H, Tien AC, Kuo CJ, Chan JR, Daneman R, Fancy SP (2016). Oligodendrocyte precursors migrate along vasculature in the developing nervous system. Science.

[bib52] Walshe TE, Ferguson G, Connell P, O’Brien C, Cahill PA (2005). Pulsatile flow increases the expression of eNOS, ET-1, and prostacyclin in a novel in vitro coculture model of the retinal vasculature. Investigative Opthalmology & Visual Science.

[bib53] Watkins TA, Emery B, Mulinyawe S, Barres BA (2008). Distinct stages of myelination regulated by gamma-secretase and astrocytes in a rapidly myelinating CNS coculture system. Neuron.

[bib54] Xiao L, Ohayon D, McKenzie IA, Sinclair-Wilson A, Wright JL, Fudge AD, Emery B, Li H, Richardson WD (2016). Rapid production of new oligodendrocytes is required in the earliest stages of motor-skill learning. Nature Neuroscience.

[bib55] Yamamuro K, Yoshino H, Ogawa Y, Makinodan M, Toritsuka M, Yamashita M, Corfas G, Kishimoto T (2018). Social Isolation During the Critical Period Reduces Synaptic and Intrinsic Excitability of a Subtype of Pyramidal Cell in Mouse Prefrontal Cortex. Cerebral Cortex.

[bib56] Yanagisawa M, Kurihara H, Kimura S, Tomobe Y, Kobayashi M, Mitsui Y, Yazaki Y, Goto K, Masaki T (1988). A novel potent vasoconstrictor peptide produced by vascular endothelial cells. Nature.

[bib57] Yeung MSY, Djelloul M, Steiner E, Bernard S, Salehpour M, Possnert G, Brundin L, Frisén J (2019). Dynamics of oligodendrocyte generation in multiple sclerosis. Nature.

[bib58] Yuen TJ, Johnson KR, Miron VE, Zhao C, Quandt J, Harrisingh MC, Swire M, Williams A, McFarland HF, Franklin RJ, Ffrench-Constant C (2013). Identification of endothelin 2 as an inflammatory factor that promotes central nervous system remyelination. Brain.

[bib59] Yuen TJ, Silbereis JC, Griveau A, Chang SM, Daneman R, Fancy SPJ, Zahed H, Maltepe E, Rowitch DH (2014). Oligodendrocyte-encoded HIF function couples postnatal myelination and white matter angiogenesis. Cell.

[bib60] Zhang Y, Chen K, Sloan SA, Bennett ML, Scholze AR, O'Keeffe S, Phatnani HP, Guarnieri P, Caneda C, Ruderisch N, Deng S, Liddelow SA, Zhang C, Daneman R, Maniatis T, Barres BA, Wu JQ (2014). An RNA-sequencing transcriptome and splicing database of Glia, neurons, and vascular cells of the cerebral cortex. Journal of Neuroscience.

